# Effect of a QTL on wheat chromosome 5B associated with enhanced root dry mass on transpiration and nitrogen uptake under contrasting drought scenarios in wheat

**DOI:** 10.1186/s12870-024-04756-8

**Published:** 2024-02-02

**Authors:** Stjepan Vukasovic, Andreas H. Eckert, Anna L. Moritz, Christian Borsch, Silvia Rudloff, Rod J. Snowdon, Andreas Stahl

**Affiliations:** 1 Department of Plant Breeding, IFZ Research Centre for Biosystems, Land Use and Nutrition, Justus Liebig University, Giessen, Germany; 2 Analytical Platform Stable Isotopes and Cell Biology, Institute of Nutritional Sciences, Justus Liebig University, Giessen, Germany; 3 Institute for Resistance Research and Stress Tolerance, Julius Kühn Institute (JKI) – Federal Research Center for Cultivated Plants, Quedlinburg, Germany

**Keywords:** ^15^N-Tracer, Nitrogen Derived from Fertilizer, Nitrogen Uptake Efficiency, Water Use Efficiency, Drought Stress Tolerance

## Abstract

**Background:**

A sufficient nitrogen supply is crucial for high-quality wheat yields. However, the use of nitrogen fertilization can also negatively influence ecosystems due to leaching or volatile atmospheric emissions. Drought events, increasingly prevalent in many crop production areas, significantly impact nitrogen uptake. Breeding more efficient wheat varieties is necessary to achieve acceptable yields with limited nitrogen and water. Crop root systems play a crucial role as the primary organ for absorbing water and nutrients. To investigate the impact of an enhanced root system on nitrogen and water use efficiency in wheat under various irrigation conditions, this study conducted two experiments using precision phenotyping platforms for controlled drought stress treatment. Experiment 1 involved four contrasting winter wheat genotypes. It included the Chinese variety Ning0604, carrying a quantitative trait locus (QTL) on chromosome 5B associated with a higher root dry biomass, and three elite German varieties, Elixer, Genius, and Leandrus. Experiment 2 compared near-isogenic lines (NIL) of the three elite varieties, each containing introgressions of the QTL on chromosome 5B linked to root dry mass. In both experiments, nitrogen partitioning was tracked via isotope discrimination after fertilization with 5 Atom % ^15^N-labeled KNO_3_^−^.

**Results:**

In experiment 1 the quantification by ^15^N isotope discrimination revealed significantly (*p* < 0.05) higher nitrogen derived from fertilizer in the root organ for Ning0604 than those of the three German varieties. In experiment 2, two out of three NILs showed a significantly (*p* < 0.05) higher uptake of N derived from fertilizer than their respective recipient line under well-watered conditions. Furthermore, significantly lower transpiration rates (*p* < 0.1) were observed in one NIL compared to its respective recipient.

**Conclusions:**

The combination of the *DroughtSpotter* facility coupled with ^15^N tracer-based tracking of N uptake and remobilization extends the insight into the impact of genetically altered root biomass on wheat NUE and WUE under different water availability scenarios. The study shows the potential for how a modified genetic constitution of the locus on wheat chromosome 5B can reduce transpiration and enhance N uptake. The dependence of the observations on the recipient and water availability suggests a need for further research to investigate the interaction with genetic background traits.

**Supplementary Information:**

The online version contains supplementary material available at 10.1186/s12870-024-04756-8.

## Background

The increasing world population and simultaneously occurring environmental changes resulting from climate change are creating an urgent need for sustainable agriculture, which will not only allow the production of sufficient food but also cause reduction in the environmental footprint of agricultural production [1, 2]. Nitrogen (N) is the most important plant nutrient used as a fertilizer in terms of quality and quantity in modern wheat production and thus essential for farmers to meet their economic goals [3]. Furthermore, N is the key nutrient for high quality baking wheat, since the expression of the protein fractions gliadin and glutenin is strongly affected by the N supply in addition to the genetic background [4, 5]. Consequently, to ensure a sufficient protein production farmers usually apply three N applications to ensure a high supply of N [6]. However, overuse of N fertilizer can also have negative environmental impacts, such as the high use of fossil fuel and the release of carbon dixoide (CO_2_) during the artificial synthesis of ammonia, which requires approximately 2% of the world's energy [7, 8]. Furthermore, direct greenhouse gas emissions resulting from ammonia and nitrous oxide are major contributors to climate change, and the leaching of nitrate contributes strongly to agroecosystem groundwater contamination [9–12]. Anthropogenically induced climate change has also increasingly impacted crop production due to intense, erratic drought stress events. Drought stress is one of the most relevant abiotic stress factors, especially in arid and semi-arid cropping regions, where it can cause yield losses ranging from 13 to 94% [13, 14]. As a result of these challenges, there is a growing interest in breeding varieties that have higher N use efficiency (NUE) and water use efficiency (WUE), thereby maintaining the required yield and quality parameters while reducing the environmental footprint [15]. NUE is defined as grain yield per unit of available N in the soil, or, concerning biomass production, as fresh matter or dry mass per unit of available N in the soil [15–17]. WUE is defined as grain yield or biomass per unit of water used [18].

NUE is a complex trait that can be described by two main components: N uptake efficiency (NupE) and N utilization efficiency (NutE). NupE describes the ability of the plant to mobilize and acquire N from the soil, whereas NutE describes the ability of the plant to translocate the acquired N into yield-producing organs (seed or biomass) [17, 19]. Especially under low N availability, high NupE is a critical variable that can most effectively increase total NUE in wheat [20, 21]. This is also in line with studies that found that NUE under low N availability is mainly explained by high NupE rather than NutE [22]. Conversely, the importance of NutE for overall NUE increases with increasing N availability, hence the NupE is particularly important in intensive cropping systems.

Roots, as the primary organ of nutrient and water uptake, are of particular significance to increase NupE and NUE for intensive wheat production systems [23–25]. For example, a root system with an increased root length density is considered to have a specifically high potential to increase NupE [26]. Particularly the mobilization of N before anthesis contributes more to N storage in the grain than remobilization after anthesis [22]. A rapid and extensive root system development in early growth stages can increase the pre-anthesis N uptake and thus increase the total NUE [27]. Therefore, it is especially important to investigate the influence of an increased root system on the mobilization (NupE) as well as the translocation (NutE), to obtain a holistic picture for the establishment of NUE. For this purpose, the use of ^15^N-labeled fertilizers is particularly advantageous to track the utilization of applied N beginning from a defined developmental stage [28].

Due to the focus on aboveground traits in crop breeding programs, it is assumed that modern elite varieties may have undergone an unintended negative selection against advantageous root traits at the cost of aboveground biomass and yield [29]. One reason for this might be the pleiotropic effects of favorable aboveground traits which have a negative effect on root characteristics. For example, negative pleiotropic effects on root traits have been demonstrated in several reduced height genes, which have played a major role in the improvement of harvest index in modern elite varieties [30]. Linkage drag may also lead to unfavorable root systems in modern elite cultivars. For example, it was found that two haplotype blocks containing quantitative trait loci (QTL) with positive effects on root dry mass (RDM) were absent in European elite wheat cultivars due to linkage drag with a QTL controlling heading date [31]. The two beneficial haplotype blocks, *Hap-5B-RDMa* and *Hap-5B-RDMb*, were found to be highly conserved and exclusively present in Chinese wheat varieties.

To date, most modern wheat breeding programs do not apply specific selection for genotypes with positive root traits, in large part due to the difficulty of phenotypic quantification [32]. One way to overcome this problem could be the use of marker-assisted selection for root-associated QTL, provided such QTL have been sufficiently tested and their positive effects validated. Here the impact of the haploblock types *Hap-5B-RDMa* and *Hap-5B-RDMb* was tested with respect to: (i) water use, (ii) N uptake and utilization, and (iii) their influence on aboveground plant organs, to offer potential solutions for practical plant breeding to improve water and nutrient use through altered root system in modern wheat lines.

## Methods

### Plant material

#### Experiment 1

In experiment 1 a set of four genotypes was investigated. These comprise the Chinese wheat variety Ning0604, which carries the two root-associated haplotype blocks *Hap-5B-RDMa* and *Hap-5B-RDMb* described in previous studies [31]. The remaining three varieties were the German elite winter-wheat varieties Elixer, Genius and Leandrus (recipients) respectively. Each elite variety corresponds to a specific wheat grain quality group. Elixer is characterized as a C-group wheat variety according to the German quality classification system, producing seeds with lower protein concentrations. Genius is listed within the E-group, which has the highest standards for protein content and quality. Leandrus is classified as a regular baking wheat variety (A-group). Leandrus carries one RDM haplotype block allele (*Hap-5B-RDMb*) but not the other, whereas the two other elite varieties do not carry either of the haplotype block alleles under investigation in this study (Table 1).

**Table 1:** Haplotype variants for *Hap-5B-RDMa* and *Hap-5B-RDMb* within the plant material used in experiment 1 alleles marked in bold letters represent the haplotype variant associated with an enhanced root growth

	**Hap-5B-RDMa**		**Hap-5B-RDMb**		
	BobWhite_c43_86		BS00029852_51	Tdurum_contig48959_1172	
Genotype	Allele	Haplotype Variant	Allele	Allele	Haplotype Variant
	[G/A]		[C/T]	[G/A]	
Elixer	GG	H1	CC	GG	h1
Genius	GG	H1	CC	GG	h1
Leandrus	**AA**	**H2**	TT	GG	h2
Ning 0604	**AA**	**H2**	**TT**	**AA**	**h3**

#### *Experiment 2*

To examine the isolated effect of the root-associated haplotype-blocks, near-isogenic lines (NIL) were developed by crossing each of the three recipients with the Ning0604 and subsequent marker-assisted backcrossing over three generations. After each backcrossing step, foreground selection on the root-associated haplotype-blocks was performed using three SNP markers associated with *Hap-5B-RDMa* and *Hap-5B-RDMb*, while background selection on the respective recipient was performed using 29 SNP markers (Table 2). For experiment 2, three NILs were selected, corresponding to the three elite recipients and carrying both haplo blocks of interest in the largest possible genetic background of each recipient. In addition, the three corresponding recipients (PAR) were also tested again, so that experiment 2 consists of six genotypes.

**Table 2:** SNP markers used for NIL development alleles. Underlined alleles represent the undesired allele frequency for the respective background

Marker	Allele	Background	Genotype		
			Elixer	Genius	Leandrus
SNP-01	[C/T]	Ning0604	T:T	T:T	T:T
SNP-02	[C/T]	Ning0604	T:T	T:T	T:T
SNP-03	[G/A]	Ning0604	A:A	A:A	A:A
SNP-04	[G/A]	Parental	A:A	A:A	A:A
SNP-05	[G/A]	Parental	A:A	A:A	A:A
SNP-06	[G/A]	Parental	G:G	G:G	G:G
SNP-07	[G/A]	Parental	G:G	G:G	G:G
SNP-08	[C/T]	Parental	C:C	C:C	C:C
SNP-09	[C/T]	Parental	C:C	C:C	C:C
SNP-10	[C/T]	Parental	T:T	T:T	T:T
SNP-11	[G/A]	Parental	G:G	G:G	G:G
SNP-12	[G/A]	Parental	A:A	A:A	A:A
SNP-13	[G/A]	Parental	A:A	A:A	A:A
SNP-14	[C/T]	Parental	T:T	C:C	C:C
SNP-15	[G/A]	Parental	A:A	A:A	A:A
SNP-16	[C/T]	Parental	C:C	C:C	C:C
SNP-17	[G/A]	Parental	A:A	A:A	A:A
SNP-18	[C/T]	Parental	C:C	T:T	T:T
SNP-19	[G/A]	Parental	A:A	A:A	A:A
SNP-20	[G/A]	Parental	G:G	G:G	G:G
SNP-21	[C/T]	Parental	C:C	C:C	C:C
SNP-22	[C/T]	Parental	C:C	C:C	C:C
SNP-23	[C/T]	Parental	C:C	C:C	C:C
SNP-24	[G/A]	Parental	A:A	A:A	A:A
SNP-25	[C/T]	Parental	C:C	C:C	C:C
SNP-26	[G/A]	Parental	A:A	A:A	A:A
SNP-27	[G/A]	Parental	A:A	A:A	A:A
SNP-28	[G/A]	Parental	A:A	A:A	A:A
SNP-29	[C/T]	Parental	C:C	A:A	C:C
SNP-30	[G/A]	Parental	A:A	A:A	A:A
SNP-31	[G/A]	Parental	G:G	G:G	G:G
SNP-32	[G/A]	Parental	A:A	A:A	A:A

### Phenotypic analysis

#### Plant cultivation and experimental setup

To gain a detailed view of WUE, transpiration efficiency, NUE as well as growth behaviour under near-field conditions, experiment 1 and experiment 2 were conducted using two different custom-built *DroughtSpotter*® precision phenotyping systems (Phenospex, Heerlen, Netherlands). The *DroughtSpotter®* is a phenotyping platform designed for drought-stress related trials using large growth containers placed on gravimetric scales, which record weight deviations every five minutes throughout the whole experiment. Every container weight scale is also individually connected to an irrigation system, allowing specific irrigation treatments for each container. The large containers enable multiple plants to be grown at field planting density to simulate field growing conditions with canopy and underground nutrient competition.

**Fig. 1:**
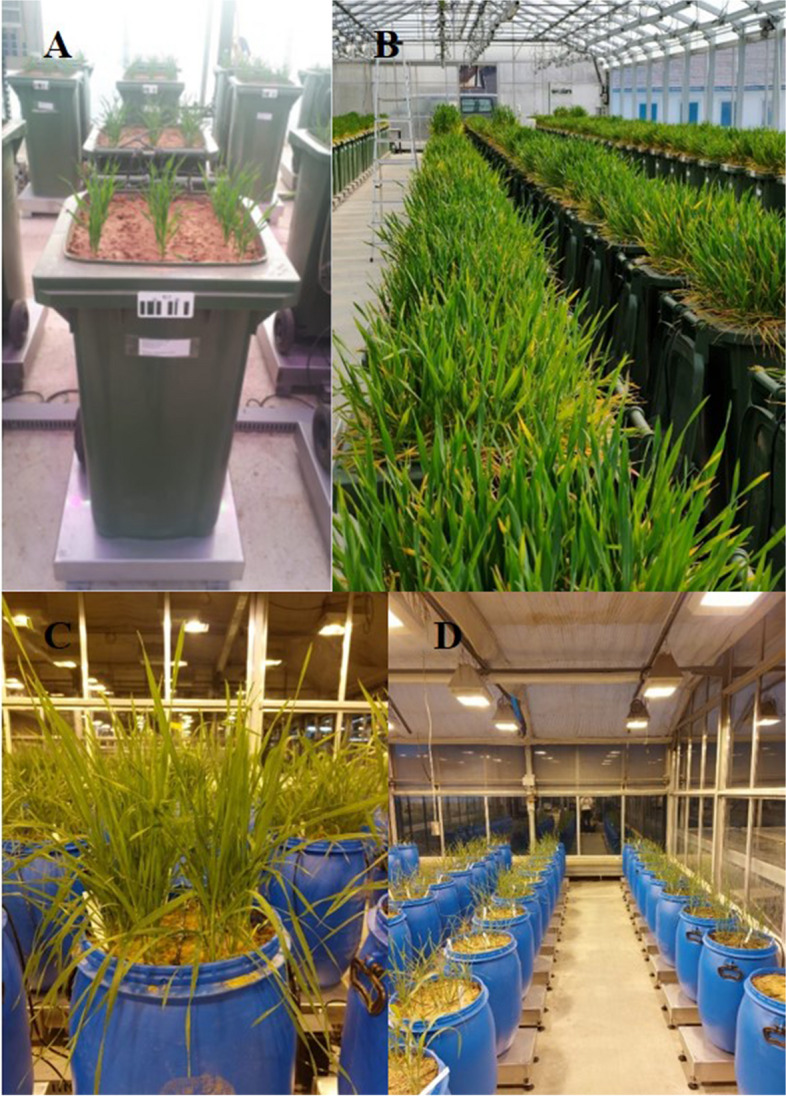
General Setup of the DroughtSpotter® phenotyping platforms located at the University of Giessen (**A**) 90L growth container placed on weights scale in *DroughtSpotterXXL* with 48 wheat plants sown in three rows á 16 plants (**B**) Impression of the full *DroughtSpotterXXL* test facility (**C**) 60L growth container placed on weight scale in *DroughtSpotterL* with 16 plants (**D**) Impression of the full *DroughtSpotterL* test facility

Experiment 1 was conducted as a full growth cycle trial using the *DroughtSpotter XXL* (DS XXL) foil house facility located at the Rauischholzhausen research facility of the Justus Liebig University Giessen in Hesse, Germany. The DS XXL is a semi-controlled phenotyping platform comprising a total of 240 large plant containers (90 L volume) placed on individual scales. Each container was filled with 150 kg of a soil mixture composed of 40% excavated soil from a local field and 60% sand to ensure sufficient drainage throughout all soil layers (Fig. 1A and B). Climate conditions including temperature (°C), relative humidity (%), and photosynthetically active radiation (µmol/m^2^) were recorded throughout the whole experiment from a weather station positioned in the center of the greenhouse. In experiment 1, the records of climate data, for the period from -84 days after Heading (DAH) until 28 DAH, show an average temperature of 13.41 °C and a mean relative humidity of 62.94%. Further information regarding the environmental conditions is given in Table 3 and Additional file 1. Containers were sown in three rows of 21 seeds per row and rows were thinned after germination to achieve a total plant density of 48 plants per container.

**Table 3:** Climatic conditions for experiment 1 and experiment 2

Experiment		Temperature [°C]	Rel. Humidity [%]	PAR [µmol/m^2^]
Experiment 1	Mean	13.41	62.94	276.13
	Median	13.8	60.31	272.69
	SD	5.15	11.54	97.10
	Min	3.42	40.85	67.54
	Max	24.22	92.78	461.62
Experiment 2	Mean	24.25	50.24	NA
	Median	25.0	49.5	NA
	SD	3.01	6.83	NA
	Min	15.0	25.5	NA
	Max	37.0	76.0	NA

In experiment 1, three different irrigation treatments were applied for each genotype. A well-watered treatment was set at 60% field capacity during the whole duration of the trial and was used as a control. Further, two drought treatments were used to investigate the drought stress resistance, varying in the date of application. The first treatment, drought scenario 1, was applied at the heading date (0 DAH) and the second treatment, drought scenario 2, was applied 14 days after heading (14 DAH). For each drought treatment, 40% field capacity was used. Each treatment was replicated three times as a fully randomized complete block design containing one replication per block.

Experiment 2 was conducted under controlled conditions using the *DroughtSpotter L* (DS L) in a greenhouse facility located at the Justus Liebig University Giessen. The DS L uses a total of 48 large plant containers (60 L volume) placed on individual heavy-duty scales. Each container was filled with 80 kg of the same soil mixture used in the DS XXL (Fig. 1C and D). Containers were sown with 21 seeds per container arranged in concentric circles and thinned after germination to achieve a total plant density of 16 plants per container. Climate conditions including temperature (°C) and relative humidity (%) were recorded using three data loggers (EL-USB-2, Lascar Electronics, Whiteparish, UK) placed throughout the greenhouse chamber. The air temperature in experiment 2 was set to 24 °C during the day and 18 °C at night, however, fluctuations in temperature could occur due to intense solar radiation, resulting in higher temperatures observed at times. In experiment 2, the records of climate data show an average temperature of 24.25 °C and a mean relative humidity of 50.24%. Further information regarding the environmental conditions is given in Table 3 and Additional file 1. Day length was set on a long day interval with 16-h days and 8-h nights. To ensure sufficient radiation, high-pressure sodium-vapour lamps (SOD Agro 400–230, DH Licht GmbH, Wülfrath, Germany) were used as soon as sunlight radiation dropped below 12 klx. Two irrigation treatments were used in experiment 2, which began 21 days after sowing (0 DAT) and were kept constant during the whole duration of the trial. A well-watered control was set at 60% field capacity and drought treatment at 40% field capacity. Each treatment was replicated three times. The experiment was laid out as a fully randomized complete block design, containing one replication per block.

### ***Use of ***^***15***^***N-Tracers***

#### Application and sample collection

In experiment 1, N was applied at the end of the booting stage (Zadoks 57–59). Each container received 2 g Nin the form of 5 atom % K^15^NO_3_^−^. Samples were taken four times at 0 DAH, 14 DAH, 21 DAH and at harvest. To avoid any edge effects plant samples were taken from the middle rows from each container and time point as well as a 15 cm deep soil sample. After the collection each sampled plant was further divided into three primary plant organs: Spike, stem (incl. leaf) and roots. A total of 576 individual samples were recorded for further analysis.

In experiment 2, N was applied 21 days after sowing (0 DAT), when the majority of the plants reached the third to fourth leaf stage (Zadoks 13–14). Each contaniner received 1 g N in the form of 5 atom % excess K^15^NO_3_^−^. Samples were taken five times at 0 DAT, 14 DAT, 28 DAT, 42 DAT and at harvest by collecting one representative plant per container and time point as well as a 10 cm deep soil sample. In experiment 2, 180 individual samples were collected for further analysis but no separation of the collected material was conducted as the sample collection was done at earlier developmental stages.

### Sample preparation and analysis

A sub-sample was taken from each sample, and transferred into 10-ml bottles along with five stainless-steal beads per bottle, to grind each sample to a fine homogenous powder using a TissueLyser II® (Qiagen, Venlo, Netherlands). Afterwards, the samples were dried at 105 °C for three hours. For the isotope-ratio mass spectrometry, 10 mg from each plant sample and 25 mg from each soil sample were weighed into tin capsules in two replications, respectively. Sample combustion was performed using a Vario PyroCube (Elementar, Langenselbold, Germany). Here, the sample was combusted at 920 °C and N was oxidized to nitrogen oxide using injected oxygen gas in abundance and helium as carrier gas. After reduction on elemental copper, the N-fraction was injected into the Isotope-ratio mass spectrometer (IRMS) using an Isoprime®-IRMS (IRMS; Elementar UK, Stockport, UK). In the IRMS, sample peaks and the appropriate reference gas peaks were ionized and the ion ratio was quantified, using a 29/28-ratio for N. Further, the reference gases were calibrated against standard samples with a known isotope-amount ratio obtained from the International Atomic Energy Agency (IAEA, Vienna, Austria). Calculations were made using the Ion Vantage® software (Elementar UK, Stockport, UK).

### Methods of calculation

N derived from fertilizer (Ndff) was calculated using Eq. 1 [28]. In this study all ^15^N values were expressed in the atom percent excess, applying a correctional factor for background abundance (0.366%).

Equation 1:

$$Ndff \left(\%\right)=\left(B-A\right)/\left(C-A\right)*100$$

where A is the ^15^N abundance of the soil at the particular measurement, B is the ^15^N atom percent excess in the plant material and C is the ^15^N atom percent excess in the applied N fertilizer.

### Ca*lculation of NUE*

In both experiments, NUE parameters were calculated at harvest according to Good et al. (2004). NUE of the grain weight (NUE_GW_) was calculated for experiment 1 and represents the quotient of the grain weight harvested per container divided by the N supplied per container. In both experiments, NUE for the straw weight (NUE_SW_) was calculated and represents the quotient of the straw weight harvested per container divided by the N supplied per container.

### Assessment of growth parameters

**Fig. 2:**
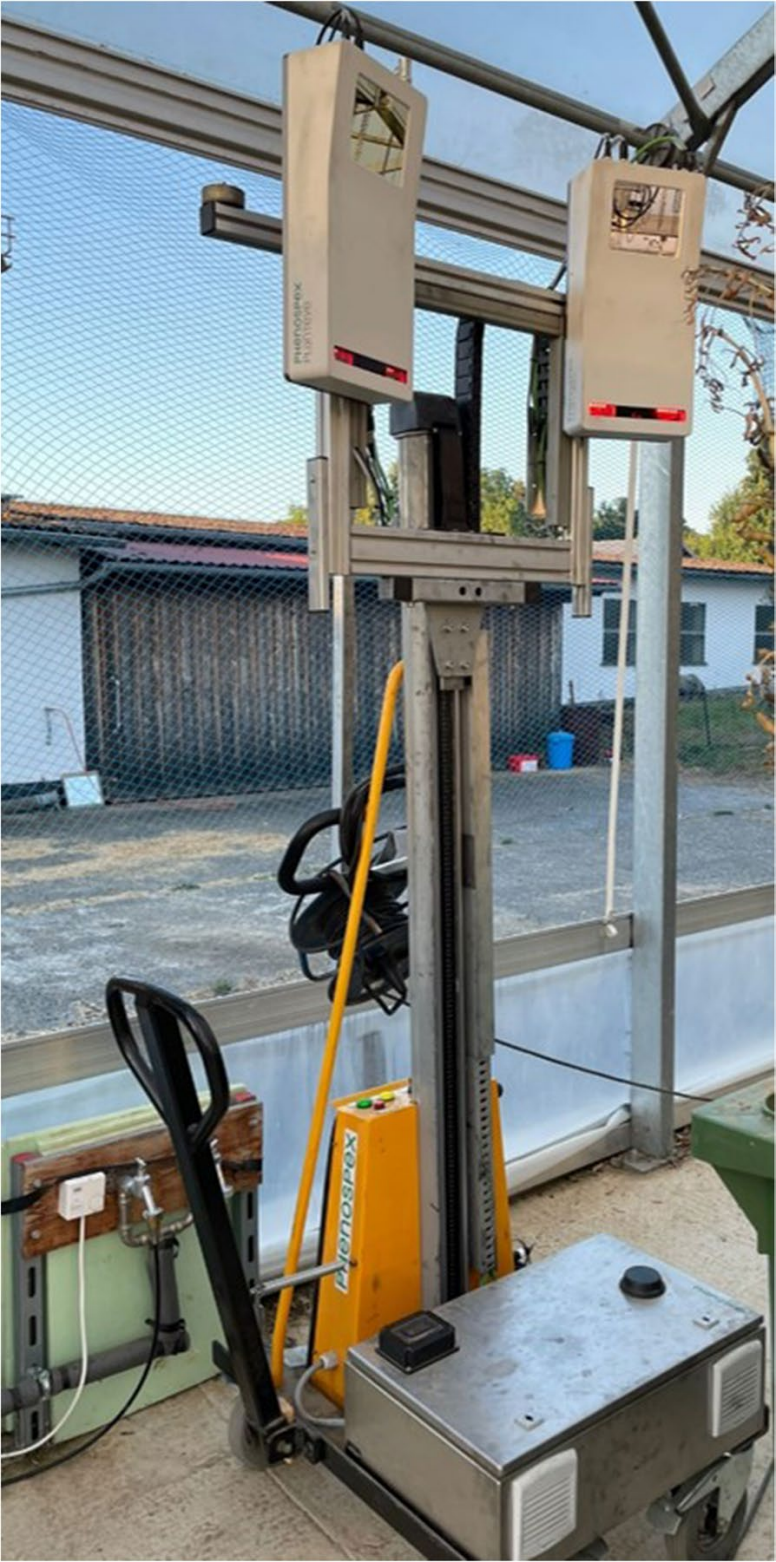
Customized version of the PlantEye® 3D-Scanner used in the DroughtSpotterXXL phenotyping platform

In experiment 1, growth parameters were recorded weekly starting with tillering (Zadoks 21) using the PlantEye F500® (Phenospex, Heerlen, Netherlands). The PlantEye F500® is a multi-spectral 3D laser scanner able to detect a wide range of relevant growth parameters and growth indices, such as digital biomass, digital height, normalized differential vegetation index (NDVI), normalized pigment chlorophyll ratio index and plant senescence reflectance index (Fig. 2).

To gain a detailed view of the physiological properties, leaf dimensions were recorded every week in experiment 2, beginning 14 days after sowing (-7 DAT). From each container, four plants were selected randomly and used throughout the whole experiment to measure the leaf characteristics by measuring length and width as well as chlorophyll content index (CCI) and leaf temperature of the youngest fully elongated leaf and the second youngest fully elongated leaf according to [33]. CCI was measured using the CCM-200 Chlorophyll Content Meter^©^ (Opti-Sciences Inc, New Hampshire, USA). The CCM-200 provides a rapid, non-destructive method for assessing chlorophyll content in leaves by applying the principles of optical absorption. The instrument uses two specific wavelengths, 653 nm in the red spectrum and 931 nm in the near-infrared spectrum. The measuring area of the device is 71 mm^2^ with a diameter of 9.52 mm. To obtain a representative value for each measurement, two technical repetitions were conducted per leaf, which were then averaged. Leaf temperature was recorded by measuring the temperature of the bottom side of the leaf using an IR 260-8S Voltcraft® infrared thermometer (Conrad Electronic, Hirschau, Germany). In both experiments, the Zadoks Growth Scale was used as the guiding scaling [34].

### Collection of yield and biomass data

In experiment 1, the aboveground biomass of each container was harvested at the ripening stage (Zadoks 91–92) and subsequently divided into two primary plant organs: spike and stem (incl. leaf). For all collected plant biomass samples, both, fresh and dry weight was recorded. Further, primary yield components such as grain yield per container, thousand kernel weight (TKW) as well and seeds per spike were analyzed. In experiment 2, both, the fresh and dry weight of the aboveground biomass of each container was recorded at the end of tillering (Zadoks 29). The remaining root material was extracted from the soil separately. Fresh and dry weight was determined for all collected plant biomass samples.

### Statistical analysis

For traits that were recorded at harvest, such as grain yield, thousand kernel weight and shoot dry mass, analysis of variance was conducted using Eq. 2. For all traits for which Eq. 2 was used, an alpha of 5% was used as the significance level. An analysis of variance was conducted using Eq. 3 for traits that were recorded over several time points during the experiment. An alpha of 5% was used as the significance level for all traits, except for daily and cumulative transpiration in experiment 2, where an alpha of 10% was used as the significance level. All comparisons reported to show significant differences were tested at a significance level of alpha 5%. For the calculation of the analysis of variance, the R-language based package lmerTest was used (http://CRAN.R-project.org/package=lmerTest). Based on the analysis of variance least significant differences were calculated using the R-language based package agricolae (http://CRAN.R-project.org/package=agricolae).

Equation 2:

$${P}_{ijlmn}=\upmu +{g}_{i}+{t}_{j}+ {W}_{l}+{C}_{m}+{R}_{n}+{e}_{ijlmn}$$

where *P*_*ijlmn*_ is the phenotypic value of the *i*^*th*^ genotype, in the *j*^*th*^ irrigation treatment, the *l*^*th*^ replication, the *m*^*th*^ column and the *n*^*th*^ row. *µ* is the overall mean, *g*_*i*_ describes the fixed effect of the *i*^*th*^ genotype, and *t*_*j*_ is the fixed *j*^*th*^ irrigation effect. *W*_*l*_ is the random effect of the *l*^*th*^ replication, *C*_*m*_ represents the random effect of the *m*^*th*^ column and *R*_*n*_ represents the random effect of the *n*^*th*^ row. The error term is represented by *e*_*ijlmn*_.

Equation 3:

$${P}_{ijklmn}=\upmu +{g}_{i}+{t}_{j}+{s}_{k}+ {W}_{l}+{C}_{m}+{R}_{n}+{e}_{ijklmn}$$

where *P*_*ijlmn*_ is the phenotypic value of the *i*^*th*^ genotype, in the *j*^*th*^ irrigation treatment, the *k*^*th*^ time point of measurement, in the *l*^*th*^ replication, the *m*^*th*^ column and the nth row. *µ* stands for the overall mean, *g*_*i*_ describes the fixed effects of the *i*^*th*^ genotype, *t*_*j*_ stands for the *j*^*th*^ irrigation treatment and *s*_*k*_ describes the *k*^*th*^ time point of measurement. *W*_*l*_ is the random effect of the *l*^*th*^ replication, *C*_*m*_ represents the random effect of the *m*^*th*^ column and *R*_*n*_ represents the random effect of the *n*^*th*^ row. The error term is represented by *e*_*ijlmn*_.

## Results

### Phenotypic characterization of drought response

#### Yield and biomass data

**Fig. 3:**
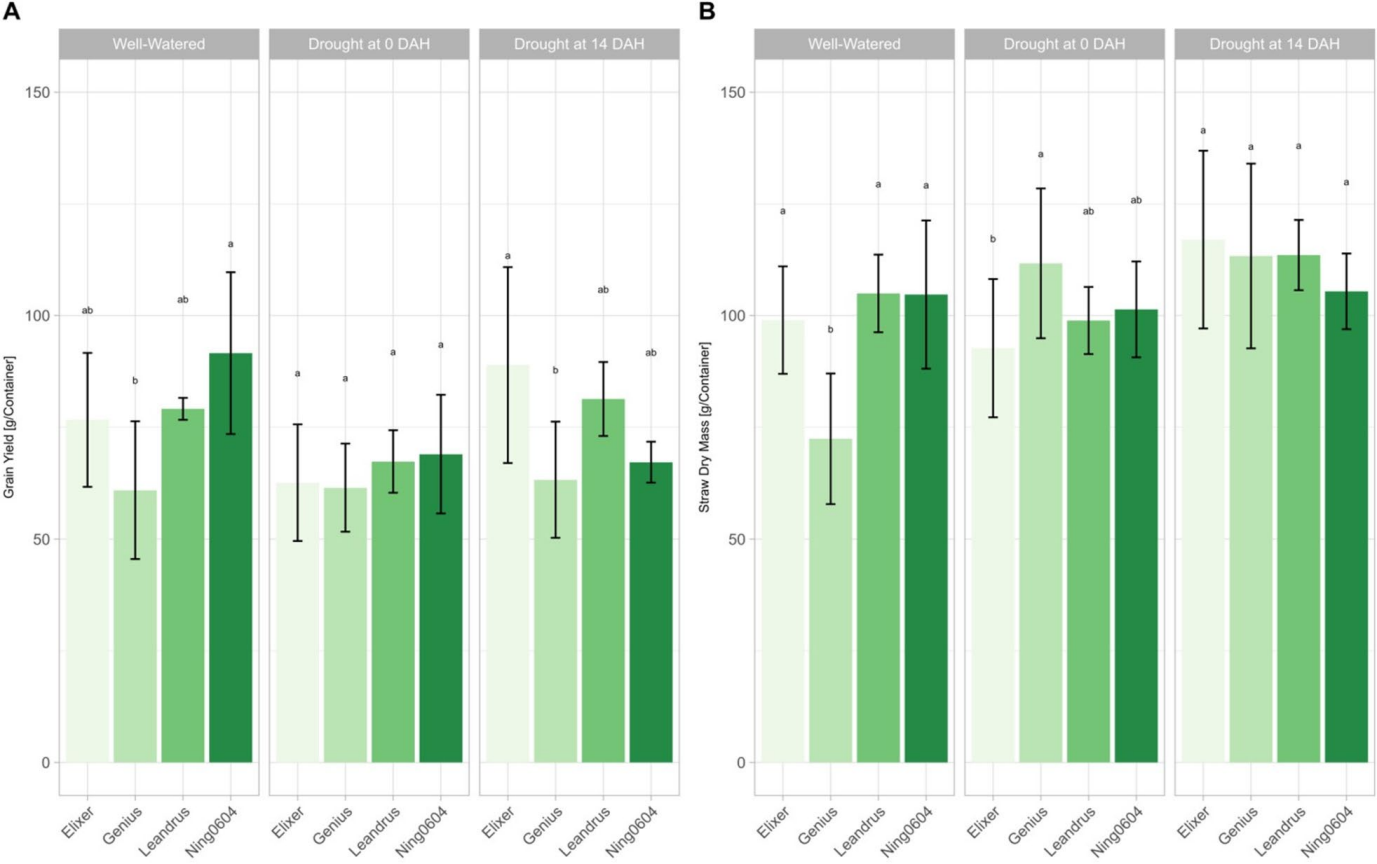
Yield and straw dry mass data of experiment 1 (**A**) Grain Yield [g/Container] under contrasting irrigation treatments (**B**) Straw dry mass [g/Container] under contrasting irrigation treatments; colours stand for different genotypes; different letters indicate significance (*p* < 0.05) between the mean values of the genotypes in the specific irrigation treatment according to the Tukey test. Error bars represent standard errors

Ning0604 completed its life cycle after 177 days from sowing until harvest while recipients required 232 days. From sowing, Ning0604 reached the heading date (HD; Zadoks 59) within 170 days, followed by Elixer and Genius, which took 195 days to develop fully emerged spikes. With 202 days, Leandrus needed the longest time to reach the heading date. Descriptive statistical parameters (minimum, maximum and arithmetic mean), variation (Var), standard deviation (SD) and coefficient of variation (CoV) for grain yield (GY), TKW, above ground dry mass and the dry mass values of the individual plant organs spike, straw and root (RDM) of experiment 1 are given in Additional file 2. The highest mean GY per container was observed for Ning0604 in well-watered treatment, which reached 91.57 g/container (Fig. 3A, Additional file 2). Further, significant genotypic differences can be observed in well-watered conditions and drought scenario 2. In all three treatment levels, Genius had the lowest GY, which was significantly lower under well-watered conditions compared to RDM donor and significantly lower than the GY of Elixer in drought scenario 2 (Fig. 3A, Additional file 2). Straw weight showed noticeably higher average values in drought scenario 2, compared to well-watered conditions, however without showing any significant differences between the genotypes. Significant differences were observed in the well-watered variant, where Genius had a significantly lower straw dry mass than the remaining three lines. Furthermore, Genius had a significantly higher straw dry mass than Elixer in drought scenario 1 (Fig. 3B, Additional file 2). For TKW, significant differences were observed in Elixer, which had the highest overall TKW in drought scenario 2 (4.01 g) and the lowest TKW in drought scenario 1 (3.38 g). The recorded biomass data revealed significant differences only for spike weight and RDM. For spike weight, the highest value can be found for Ning0604 in well-watered conditions with 125.90 g. For RDM, the highest value was found for Elixer in drought scenario 2 with 192.33 g (Additional file 2).

Descriptive statistics for experiment 2 can be found in Additional file 3. No significant differences were detected for dry mass, but as expected higher dry mass values were seen for all genotypes in the well-watered treatment. For RDM no significant differences between genotypes or treatment were observed. Furthermore, for all recorded traits, no significant differences were found between the recipient and the NILs of the respective genotype. For fresh matter content, the highest values were achieved by Genius NIL under well-watered conditions, for dry mass the highest value was recorded for Elixer PAR in well-watered treatment (Additional file 3).

### Above ground growth parameters

In experiment 1, the weekly PlantEye measurements of growth parameters digital biomass, digital height and NDVI revealed significant differences for digital biomass in Ning0604 compared to the three elite varieties across all treatments, beginning from the first measurement at -14 DAH until harvest (Additional file 4). A similar pattern was observed for digital height, where Ning0604 showed significantly higher plant height across all the treatments than the three recipients (Additional file 4). NDVI in experiment 1 revealed a distinct difference in the growth behaviour of Ning0604 compared to the recipients. Under well-watered conditions in drought scenario 2, Ning0604 showed significantly higher NDVI values than the recipients until 14 DAH, but its NDVI declined more strongly from 14 DAH until harvest. Interestingly, in drought scenario 1 this behaviour was not observed (Additional file 4). Leaf area, CCI and leaf temperature measurements in experiment 2 showed no significant differences (Additional file 5).

### Analysis of gravimetrical data on water use parameters

To determine the impact of an increased root system on water use, we used a gravimetric approach in both experiments to measure daily and cumulative transpiration as well as WUE. In experiment 1, daily and cumulative transpiration were recorded from the beginning of tillering (Zadoks 21) until harvest (Zadoks 92). In experiment 2, the three recipients as well as their respective NILs were tested under the same conditions to measure the isolated effect of the root-associated haplotype blocks on daily and cumulative transpiration as well as WUE. Daily and cumulative transpiration were recorded throughout the entire duration of the experiment.

### Cumulative and daily transpiration

**Fig. 4:**
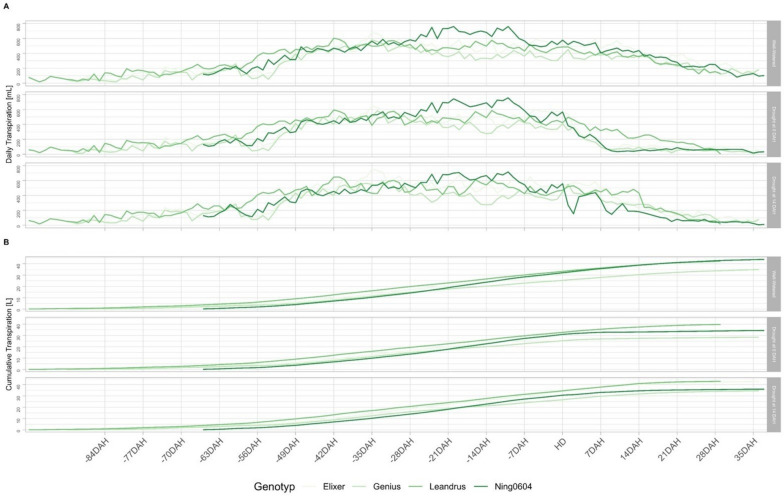
(**A**) Daily transpiration [mL] and (**B**) Cumulative transpiration [L] under contrasting irrigation treatments in experiment 1. Time line is normalized around begin heading date (0 DAH) which corresponds as treatment begin; colours stand for different genotypes

Highly significant (*p* < 0.001) differences can be observed for daily transpiration within treatment levels as well as across treatment levels. The highest daily transpiration rates for these two treatments were reached by Ning0604, which transpired 734 ml under well-watered conditions and 689 ml in drought scenario 1. For drought scenario 2, the highest transpiration rate (749 ml) was recorded for Elixer (Fig. 4). Between 84 and 49 DAH, significant differences in daily transpiration were observed between the genotypes in all three treatments. However, none of the genotypes tended to have a higher daily transpiration rate over the full course of two weeks (Fig. 4, Additional file 6). From -28 DAH until -14 DAH the Ning0604 showed significantly higher daily transpiration rates than the recipients in well-watered and drought scenario 1. A significantly higher daily transpiration rate for Ning0604 was observed from -7 DAH until HD in well-watered conditions as well as in drought scenario 1 (Fig. 4A, Additional file 6). The highest overall cumulative transpiration rate (42 L) was achieved by Elixer, Leandrus and Ning0604 in well-watered conditions, and by Elixer and Leandrus in drought scenario 2. The lowest cumulative transpiration under well-watered conditions was achieved by Genius, which had significantly lower transpiration compared to the other three genotypes from -21 DAH until harvest. In line with these findings, also in experiment 2 Ning0604 (35 L) and Genius (34 L) showed the lowest cumulative transpiration (*p* < 0.05). Further, significant differences in cumulative transpiration were observed from -84 DAH until -35 DAH. However, Leandrus had either the highest values or was at least in the same significance group as the genotype with the highest cumulative transpiration on the respective measurement day across all three treatments (Fig. 4B, Additional file 6). Except for the low transpiration of Genius under well-watered conditions, no significant differences were observed between -21 DAH and 14 DAH (Fig. 4B, Additional file 6).

**Fig. 5:**
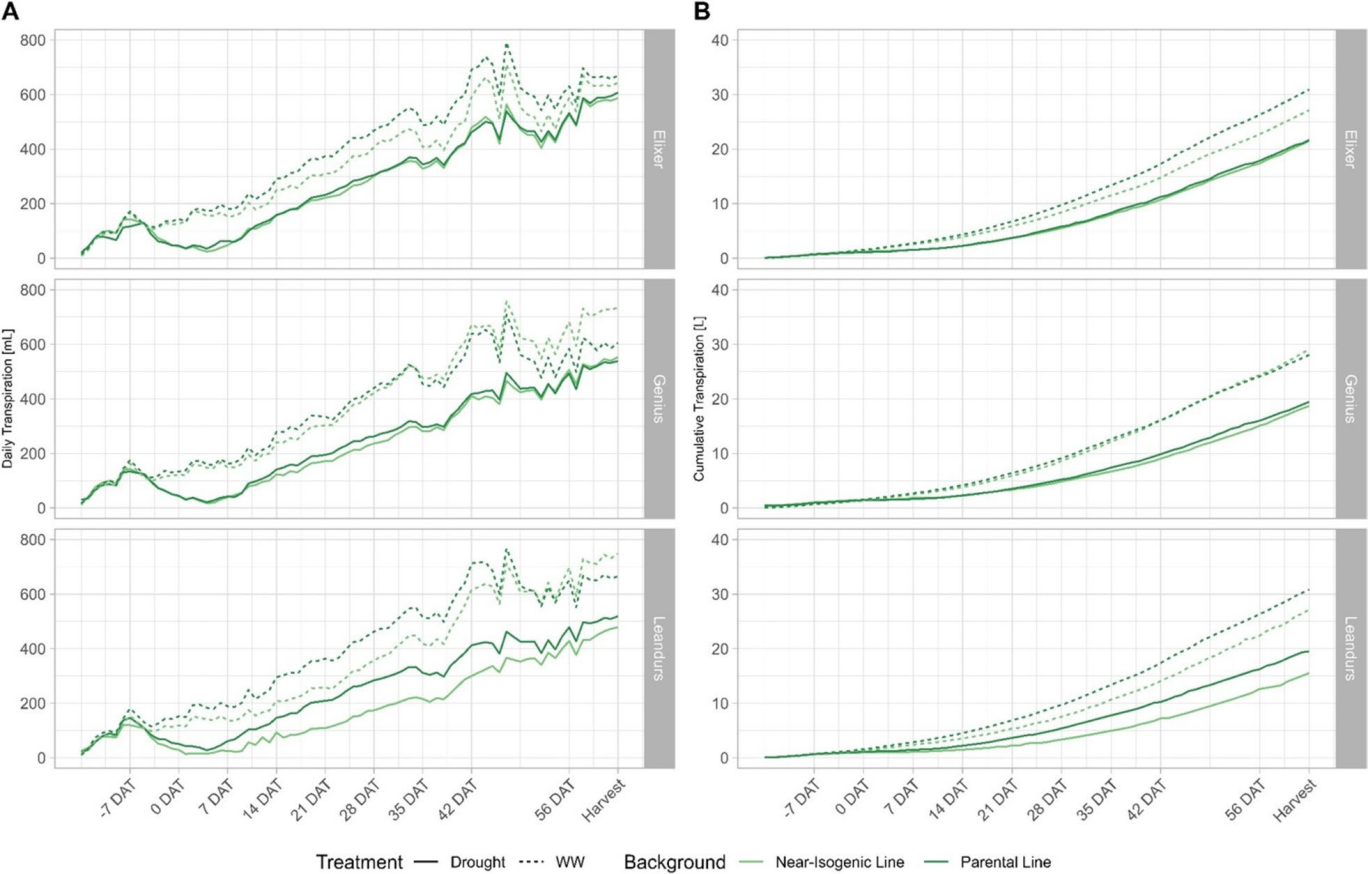
(**A**) Daily transpiration [mL] and (**B**) Cumulative transpiration [L] of the different genotypes in experiment 2. Time line is normalized for the treatment begin (0 DAT). Different line types represent the genetic background; different colours stand for the different irrigation treatment

Throughout experiment 2, significant (*p* < 0.1) differences were detected in daily and cumulative transpiration for Elixer (Fig. 5). Under drought conditions Elixer PAR showed a higher cumulative transpiration from 21 DAT until harvest than Elixer NIL. Furthermore, observation revealed lower transpiration of Leandrus NIL than Leandrus PAR under well-watered and drought conditions (Fig. 5B, Additional file 5). Similar to the cumulative transpiration, significant (*p* < 0.1) differences were found for the daily transpiration of Elixer under well-watered conditions. Here, Elixer NIL had lower daily transpiration between 0 and 21 DAT (Fig. 5A). Leandrus NIL recorded the highest daily transpiration rate under well-watered conditions (748.38 ml). A similar pattern was seen for the cumulative transpiration, which showed a continual gradient without reaching a plateau at any time. with the highest total value achieved by Leandrus PAR under well-watered conditions.

### *Water use efficiency*

**Fig. 6:**
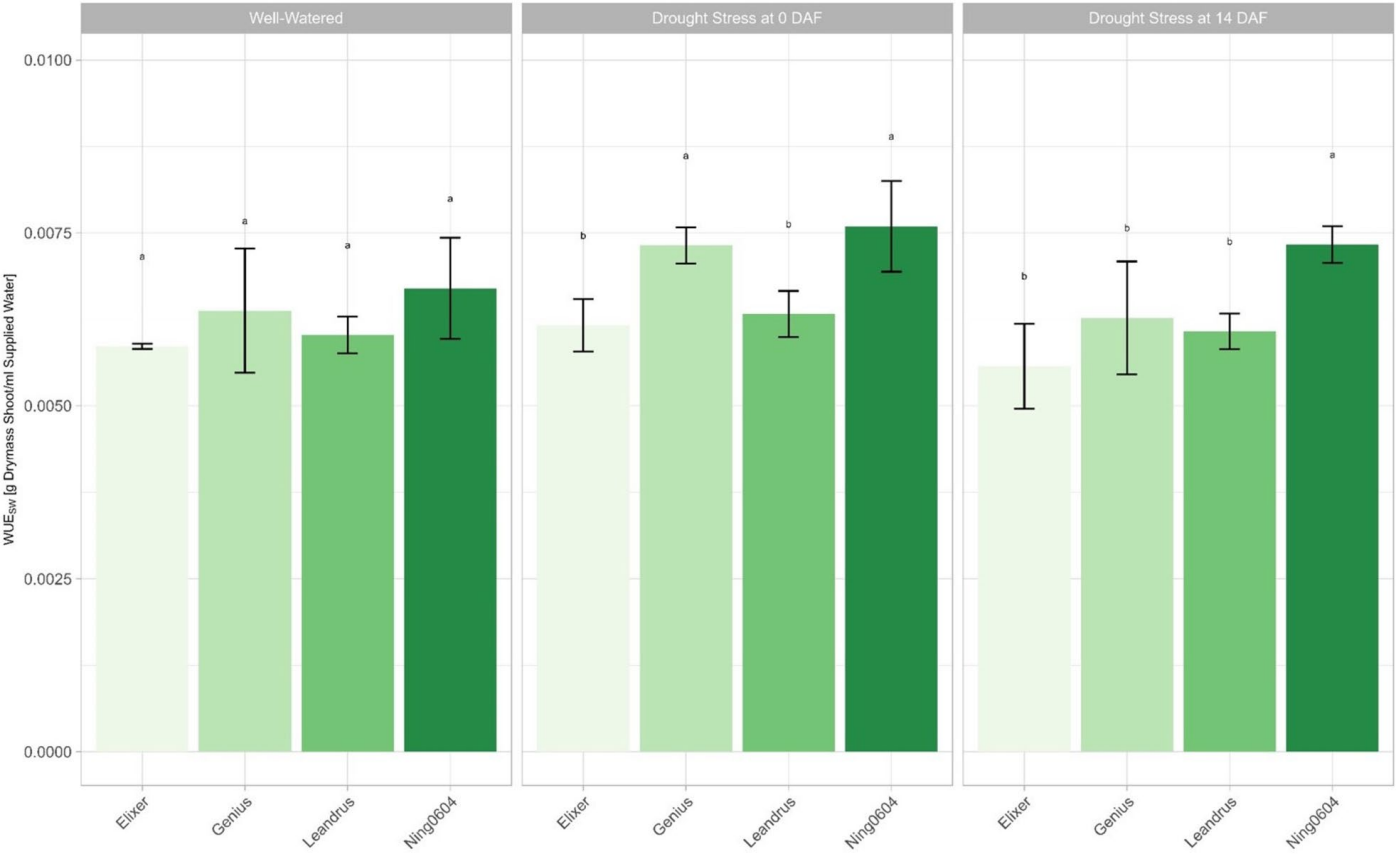
Water use efficiency [g dry mass shoot/ ml supplied water] of shoot dry mass in experiment 1 under contrasting irrigation treatments Colours stand for different genotypes; different letters indicate significance (*p* < 0.05) between the mean values of the genotypes in the specific irrigation treatment according to the Tukey test. Error bars represent standard errors

Ning0604 achieved the highest WUE in all three treatments in experiment 1. In drought scenario 1, Ning0604 had the highest WUE of 7.5 mg dry mass shoot/ml of water supplied and thus had a significantly higher WUE than Elixer and Leandrus. Genius achieved a WUE of 7.3 g dry mass shoot/ml of water supplied in drought scenario 2 and thus was at the same significance level as Ning0604 (Fig. 6). Also, under drought scenario 2, Ning0604 had the highest WUE with 7.3 mg dry mass shoot/ml water supplied and thus had a significantly higher WUE than the three German elite varieties (Fig. 6). In comparison to drought scenarios 1 and 2, no significant differences could be observed in WUE under well-watered conditions. Here, Ning0604 achieved a WUE of 6.6 mg dry mass shoot/ml supplied water (Fig. 6). The WUE measured in experiment 2 showed no significant differences between the parental lines and the NILs in either treatment. However, genotype-treatment interaction for Elixer and Genius was observed (Additional file 7).

### Phenotypic characterization of N uptake and N use

#### Nitrogen derived from fertilizer

**Fig. 7:**
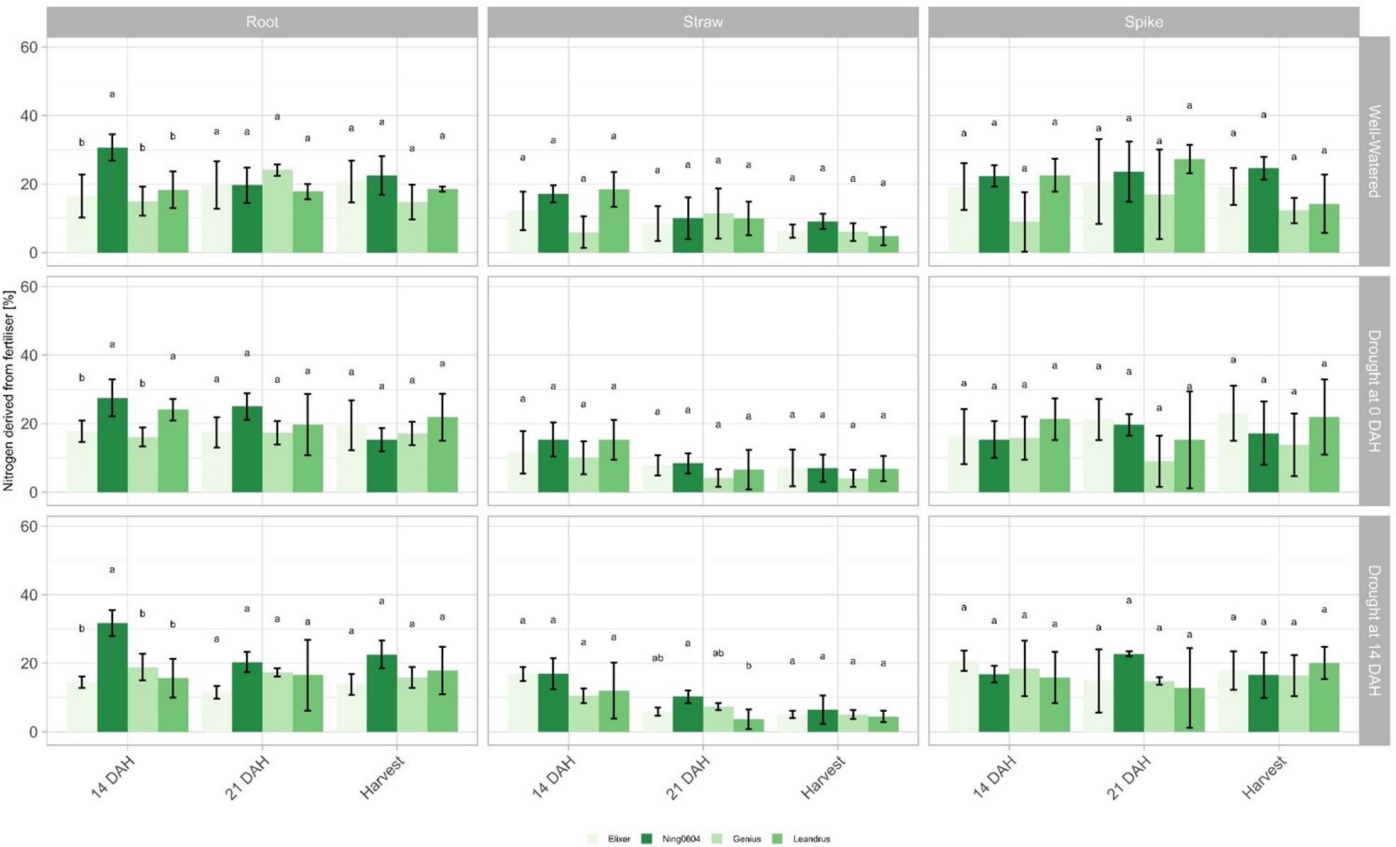
Nitrogen derived from fertiliser in different plant organs and under contrasting irrigation treatments in experiment 1. Colours stand for different genotypes; different letters indicate significance (*p* < 0.05) between the mean values of the genotypes in the specific irrigation treatment and plant organ according to the Tukey test. Error bars represent standard errors

In both experiments, 5 Atom % K^15^NO_3_^−^ was applied to calculate Ndff in order to quantify the amount of N taken up and translocation from the timepoint of application (heading date). In both experiments, Ndff showed significant differences between and within the treatments. Furthermore, significant differences were observed in Experiment 1 between the different sampling time points and between the investigated plant organs. In all three treatments, Ning0604 showed the highest Ndff values in roots at 14 DAH. Especially in well-watered conditions and in drought scenario 2, Ning0604 also showed significantly higher N uptake (30%) compared to the three recipients, which did not exceed 20% at this time point. In drought scenario 1, the Ning0604 and Leandrus had significantly higher values compared to Elixer and Genius. This is particularly noteworthy since Leandrus is the only genotype of the three elite varieties that carries one of the two root-associated haplotype blocks (Fig. 7). In all three treatments all genotypes showed a decrease in straw Ndff from 14 DAH until harvest. As for 14 DAH in roots, the Ning0604 showed constant Ndff values of 15–17% in all three treatments, indicating a lower interaction between N-translocation and water availability. This tendency was also observed in the straw at 21 DAH, where Ning0604 showed constant Ndff values of 8–10%, compared to a decrease in the three elite lines. The straw Ndff at 21 DAH in drought scenario 2 was particularly noteworthy, with Ning0604 showing significantly higher values (Fig. 7). For Ndff in spikes, no significant genetic differences were observed in any treatment or timepoint (Fig. 7).

**Fig. 8:**
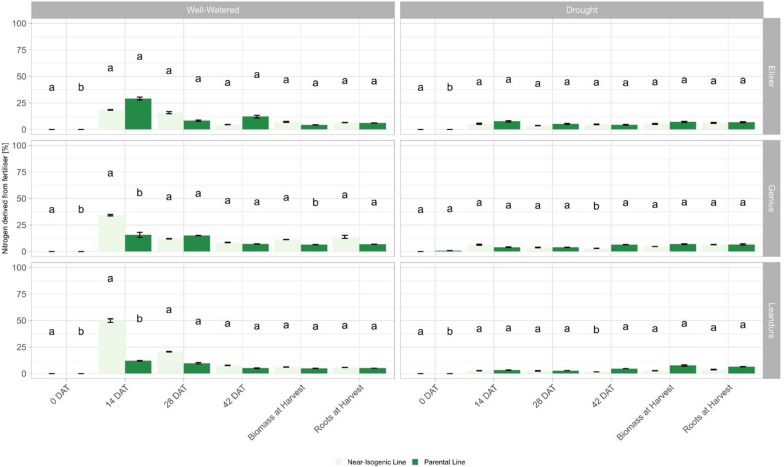
Nitrogen derived from fertiliser in different plant organs and under contrasting irrigation treatments in experiment 2. Colours represent the genetic background; different letters indicate significance (*p* < 0.05) between the mean values of the genotypes in the specific irrigation treatment according to the Tukey test. Error bars represent standard errors

Under well-watered conditions, comparable results were observed for Ndff in roots in experiment 1 at 14 DAH and 14 DAT in experiment 2. For two of the three elite varieties, the NIL showed significantly higher Ndff values than its respective recipient. In particular, the Ndff of Leandrus NIL reached only 12%, whereas the Leandrus NIL containing the Ning0604 Donor QTL exceeded Ndff values of 50% (Fig. 8).

### Nitrogen use efficiency

The overall capacity of genotypes to translocate N was determined as the NUE_GW_ and NUE_SW_ in experiment 1 and as NUE_SW_ in experiment 2, using calculations according to Good et al. (2004). In experiment 1, NUE_GW_ revealed a significantly higher NUE of Ning0604 under well-watered conditions and a significantly higher NUE for Elixer under drought scenario 2. In drought scenario 1, no significant difference was found between the genotypes. Here, Ning0604 reached the highest NUE (Additional file 8A). Furthermore, genotype-by-treatment interactions were observed. For example, Leandrus NIL showed the highest NUE under well-watered conditions but not under drought stress.

NUE_SW_ revealed a significantly higher NUE under well-watered conditions in Elixer and Ning0604 compared to Genius, which exhibited the lowest NUE under well-watered conditions (Additional file 8B). No significant differences were found between the recipients and their respective NIL for NUE_SW_. However, in well-watered conditions, the genotypes showed higher variation in NUE than in drought scenario 1, where NUE was similar in all genotypes between the NILs and their respective recipients. Furthermore, genotype-treatment interactions were identified, with Leandrus NIL showing an increased NUE under well-watered conditions while its recipient in both treatments showed constantly lower NUE. In comparison to Leandrus, an inverse behavior was seen in Genius, wherein the trend, but not significantly a higher NUE of the recipient was observed under well-watered conditions (Additional file 9).

## Discussion

### QTL x background interaction potentially affects water-uptake

Several studies have reported improvements in water uptake ability and water use efficiency, as well as yield benefits, concerning root characteristics [2, 35, 36]. It was reported that the root angle and the number of seminal roots were found to be good indicators to select root systems that show good adaptive properties to drought stress conditions and thus can have a positive impact on yield performance [37]. A well-known example of a gene influencing root growth which also provides yield benefits under water-limiting conditions is *DeepRooting1*, described in rice [38]. The importance of root systems specifically adapted to certain environmental conditions such as soil texture and water availability has been frequently described [23, 24, 39, 40]. For example, it was reported that a 34% increase in grain size can be expected by using deep-rooted genotypes under limited water availability [23]. In the present study, we hypothesized that haplotype blocks associated with enhanced root biomass have a beneficial effect on WUE under different irrigation regimes. By marker-assisted backcrossing the root-associated haplotype-blocks were introgressed into three elite winter wheat backgrounds to compare NILs with their respective recipient lines and QTL donors under controlled drought-stress scenarios. One of three NILs carrying *Hap-5B-RDMa* and *Hap-5B-RDMb* showed lower cumulative transpiration than its respective parent under well-watered conditions. The fact this phenomenon was only observed in one background indicates an interaction between the genomic background provided by the parental line and the root-associated haplotype-blocks is present. Such QTL x background interaction was also observed in similar experiments, where certain NILs showed significantly higher grain yield than their parental line in both control and stress treatments, while other NILs showed lower grain yields in both treatments [41]. Multiple studies have reported the negative pleiotropic effect of introgressed genes on yield performance across changing environments [41, 42]. Under drought conditions, no differences between NILs and their donors had been observed for cumulative transpiration, daily transpiration or shoot WUE, which excludes the negative effects of the introgressed haplotype blocks on transpiration performance or WUE. The three NILs all showed equivalent RDM values to their common QTL donor in all treatments of both experiments. Although no conclusions can be drawn regarding water uptake based on RDM alone, our results indicate that *Hap-5B-RDMa* and *Hap-5B-RDMb* can increase WUE in a genotype-dependent manner. Thus, this study suggests closer investigations for root morphology or root growth behaviour and their impact on WUE, since the DroughtSpotter phenotyping platform is limited for the investigation of such traits. Further, since this study focused on phenotypic response on WUE, the insights in this study are exclusively drawn on morpholocial an physiological characteristics at the plant level. Several molecular studies have evaluated the regulation of root associated genes and their regulation under drought conditions in wheat [43–45] and barley [46]. Although the crucial role of molecular studies in understanding the underlying mechanisms is recognized, it is worth noting the limited molecular information on the influence of *Hap-5B-RDMa* and *Hap-5B-RDMb* on the regulation of drought tolerance genes. In this context it would be of particular interest to investigate the influence of *Hap-5B-RDMa* and *Hap-5B-RDMb* on the enzymatic activity on possible effects of antioxidant activity on drought stress tolerance [47–49].

### ***Root associated haploblock-types affect Nitrogen derived from fertilizer***

The second objective of this study was to investigate the effect of *Hap-5B-RDMa* and *Hap-5B-RDMb* on NUE in general and on NupE and NutE in particular. Since the root is the primary organ for nutrient uptake, several studies have investigated the effect of root-associated traits on NUE [50–54]. Accordingly, it is well known that the N accumulated before anthesis is the main source of grain N. Hence, genetic variation in grain yield and grain N content is mainly explained by pre-anthesis N accumulation rather than post-anthesis N remobilization [22]. Increased Ndff may indicate an increased NupE before anthesis, and corresponding to this assumption we found that NILs carrying *Hap-5B-RDMa* and *Hap-5B-RDMb* exhibited higher Ndff than their respective recipients under well-watered conditions. In both hexaploid and tetraploid wheat, diverse studies have addressed the question of whether NupE or NutE contributes more strongly to improving NUE and described genetic variation for NupE and NutE [22, 55–59]. In a two-year experiment using three N levels, it was reported that 62–70% of the genetic variation for NUE was explained by NupE [58]. Comparable results were reported by other studies, where 54–63% of NUE was explained by NupE [55, 57]. The lack of significant genetic-environmental interactions in this study may be put into perspective by other, more recent studies, which found that the influence of NupE and NutE on NUE depended on soil N availability [59]. Under low and high N availability, NupE and NUE correlated significantly with each other, while NutE showed significant effects on NUE only under high N availability [57]. Since this causes NupE to exert a strong influence on NUE at several N levels, a higher importance was assigned to NupE [59]. In contrast, other studies found interactions in NUE between different N levels, but a higher significance of NutE [58]. This is consistent with studies, which reported a variation of NUE under low-N conditions being largely described by NutE [22]. Similar results were reported for maize, where the genetic variation of NUE under high-N conditions was mainly described by NupE, whereas NUE variation was mainly described by NutE under low-N conditions [60]. In both of our experiments, positive effects on Ndff were detected by genotypes carrying the haplotype blocks *Hap-5B-RDMa* and *Hap-5B-RDMb*. Furthermore, the results indicate that *these haplotype blocks* are particularly beneficial under well-watered conditions. In addition, the donor lines also show increased Ndff values in drought scenario 2, where sufficient water was presumably available at 14 DAH (Fig. 7). Two out of three NILs (Genius and Leandrus) also showed higher Ndff than their respective recipient lines under well-watered conditions. Thus, as with the previously described relationship between *Hap-5B-RDMa* and *Hap-5B-RDMb* and the transpiration parameters, QTL interactions with the genetic background of the recipient lines were also observed for the well-watered variant in combination with Ndff as the NIL of Elixer shows lower Ndff than its parental line (Fig. 8).

### Hap-5B-RDMa and Hap-5B-RDMb have no pleiotropic effect on pre-anthesis growth parameters

The third aim of this study was to test whether *Hap-5B-RDMa* and *Hap-5B-RDMb* have an impact on above ground growth characteristics Several previous studies have shown significant effects of QTL for root architecture and root growth on aboveground growth behaviour [37, 59]. In contrast, no effects of *Hap-5B-RDMa* and *Hap-5B-RDMb* on aboveground biomass were detected. This is in line with similar studies that also failed to detect significant correlations between root and shoot ratios [62]. The lack of influence on aboveground growth factors rules out the pleiotropic effects of *Hap-5B-RDMa* and *Hap-5B-RDMb* on shoot biomass. Also, drought-induced changes in root development or RDM that have been reported in other studies [20, 63] could not be confirmed. Several studies also reported positive effects of root traits on grain yield [61, 62, 64]. Unfortunately, the container size of the *DroughtSpotter L* platform used for experiment 2 is insufficient to obtain meaningful grain yield estimates, hence, we only investigated the vegetative growth phase in experiment 2, and at this stage are unable to conclude the potential effects of *Hap-5B-RDMa* and *Hap-5B-RDMb* on grain yield, or other traits determining agronomic performance in field conditions. Future experiments are required to enable more insight into these relationships.

### Implications for further studies

The results presented here show that ^15^N tracers, in combination with high-resolution phenotyping platforms, can be effectively exploited to study N uptake and N translocation under different, defined water availability conditions. In terms of N translocation, it is particularly important to consider changing relationships between sink and source during plant development. Thus, the N utilization must be considered in relation to the metabolization rate of the source organ [65]. Especially during post-anthesis, when the metabolization rates of the yield-determining organs increase, the leaf tissue serves as a valuable N source. In this study, this process was visible as a gradual reduction of Ndff in straw and leaf tissues, respectively, which are a major source of N during senescence. This finding is consistent with studies that described remobilization into the yield-producing organs as an important factor for high grain yields [66]. Various other studies also indicated the importance of high N remobilization for high grain protein content [67, 68].

Another advantage of the setup used in experiment 1 is that it allowed a detailed investigation of genotypic differences in N uptake after anthesis, which is reported to have a particularly high influence on the grain protein deficit [68–70]. One hypothesis concerning the physiological mechanisms underlying genotypic variability for this trait is that improved root penetration of the soil leads to improved N uptake [70]. Since Ning0604 and two out of three NILs showed higher Ndff, our study supports this hypothesis. This study extends insights into the impact of genetically altered root biomass on wheat NUE and WUE under different water availability scenarios. They also enabled the identification of relationships with other key traits, such as biomass. The use of ^15^N tracers for tracking N uptake and remobilization in a large-container precision phenotyping system proved to be a proficient method to measure WUE, NUE and their interaction with above ground biomass with a high resolution under field-like growth conditions. In particular, the ability to assess the uptake of applied N under different irrigation treatments, concerning the impact of root traits on N translocation highlights the value of the experimental setup. Nevertheless, potential interactions due to environmental and developmental factors must be clarified for a better understanding, since factors such as the timing and rate of N application, type and induction of drought stress as well as crop management factors such as sowing may also impact NUE and WUE. This study conducted a full growth cycle trial only in experiment 1, and since no NILs were used in this particular trial the full understanding of the impact of *Hap-5B-RDMa* and *Hap-5B-RDMb* on yield performance is still limited. Hence, two approaches to validate the effect of *Hap-5B-RDMa* and *Hap-5B-RDMb* on yield performance were conducted. First, full growth-cycle experiments in high-resolution phenotyping platforms will provide more detailed information on N uptake and N translocation under different irrigation treatments and potentially help identify suitable target environments for genotypes with enhanced root biomass. Further, although the design of the DroughtSpotter system enables a simultaneous and continuous examination of makro physological traits, we recommend the additional examination of micro physiological traits, such as stomatal aperture, to obtain higher resolutions on trait characteristics and interactions. Secondly, field trials of the NILs and their respective recipients will enable a more detailed evaluation of their performance in diverse environments. Meanwhile, the initial results presented here suggest that introgression of QTL conferring altered root biomass can be a rapid, useful and valid approach to improve NUE in wheat breeding programs.

## Conclusions

The manuscript presents a combination of unique, innovative gravimetric phenotyping platforms to assess transpiration throughout the growing season, and 15N tracers to study nitrogen uptake and translocation. It was shown that the previously identified QTL on chromosome 5B, which is associated with a larger root system, alters both transpiration and nitrogen uptake and is therefore a potential target for breeding more efficient varieties. However, there is also an interaction with the background of the respective genotypes and the specific drought scenario. Therefore, future studies should investigate the interaction of the locus with divergent environmental scenarios and potential epistatic effects in different genetic backgrounds.

### Supplementary Information

**Additional file 1. **Mean temperature [°C] and mean relative humidity [%] for experiment 1 and experiment 2.

**Additional file 2. **Descriptive statistics for collected harvest data in experiment 1.

**Additional file 3. **Descriptive statistics for collected harvest data in experiment 2

**Additional file 4. **Digital growth parameters recorded in experiment 1 using the PlantEye F500® multi-spectral 3D laser scanner

**Additional file 5. **Descriptive statistics for transpiration data as well as physiological growth parameters recorded in experiment 2 from -7 DAT until harvest

**Additional file 6. **Descriptive statistics for cumulative transpiration [L] and daily transpiration [ml] recorded in experiment 1 from -84 DAH until 35 DAH

**Additional file 7. **Water use efficiency calculated for experiment 2.

**Additional file 8. **Nitrogen use efficiency of grain weight and shoot drymass under contrasting irrigation treatments in experiment 1.

**Additional file 9. **Nitrogen use efficiency of shoot dry mass under contrasting irrigation treatments in experiment 2.

## Data Availability

The data supporting the findings of this study are available from the corresponding author, Stjepan Vukasovic (Stjepan.Vukasovic@agrar.uni-giessen.de), upon request.

## Abbreviations

CCI: Chlorophyll content index
CoV: Coefficient of variation
DAH: Days after heading date
DAT: Days after treatment begin
DS L: DroughtSpotterL
DS XXL: DroughtSpotterXXL
GY: Grain yield
HD: Heading date
IRMS: Isotope-ratio mass spectrometer
Ndff: Nitrogen derived from fertilizer
NDVI: Normalized differential vegetation index
NIL: Near-Isogenic Line
NUE: Nitrogen use efficiency
NupE: Nitrogen uptake efficiency
NutE: Nitrogen utilization efficiency
PAR: Parental line/Recipient
QTL: Quantitative trait locus
RDM: Root dry mass
SD: Standard deviation
TKW: Thousand kernel weight
Var: Variation
WUE: Water use efficiency
