# Supplementary material for: Effect of a QTL on wheat chromosome 5B associated with enhanced root dry mass on transpiration and nitrogen uptake under contrasting drought scenarios in wheat
Source: BMC Plant Biol. 2024 Feb 2;24:83. doi: 10.1186/s12870-024-04756-8 (PMC10835935; doi:10.1186/s12870-024-04756-8)
Supplement: Supplementary file 1 — Additional file 1. Mean temperature [°C] and mean relative humidity [%] for experiment 1 and experiment 2. [file 12870_2024_4756_MOESM1_ESM.docx]

**Supplementary Information**


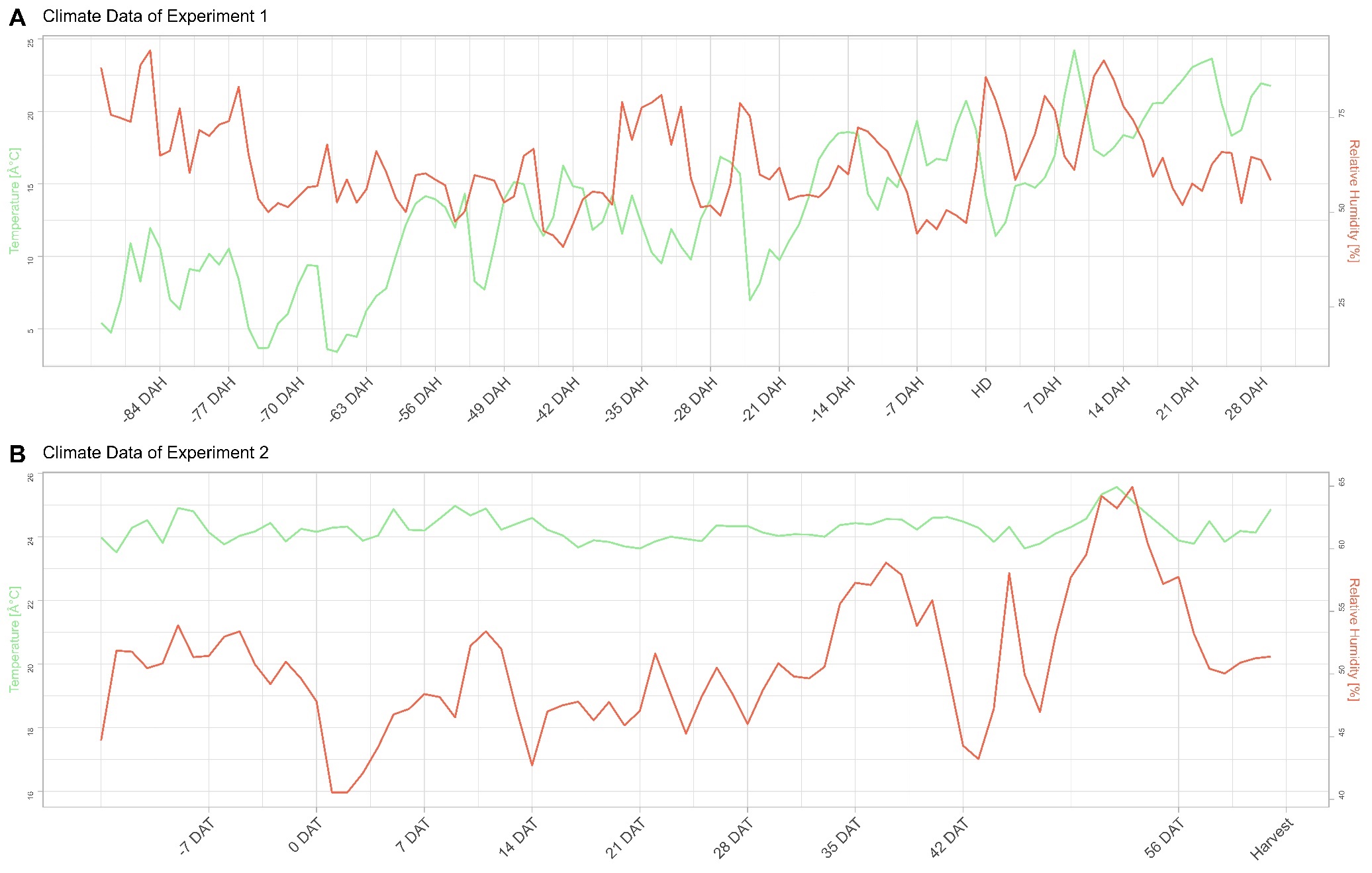


Additional file 1: Mean temperature [°C] (green graph) and mean relative humidity [%] (red graph) for experiment 1 and experiment 2. Time specifications for experiment 1 are normalized around the heading date and are specified as days after heading (DAH). Time specifications for experiment 2 are normalized around the treatment begin and are specified as days after treatment begin (DAT).
