# Supplementary material for: Effect of a QTL on wheat chromosome 5B associated with enhanced root dry mass on transpiration and nitrogen uptake under contrasting drought scenarios in wheat
Source: BMC Plant Biol. 2024 Feb 2;24:83. doi: 10.1186/s12870-024-04756-8 (PMC10835935; doi:10.1186/s12870-024-04756-8)
Supplement: Supplementary file 2 — Additional file 2. Descriptive statistics for collected harvest data in experiment 1. [file 12870_2024_4756_MOESM2_ESM.docx]

Additional file 2: Descriptive statistics for collected harvest data in experiment 1

| Trait | Treatment | Well-Watered | | | |  | Drought Scenario 1 | | | |  | Drought Scenario 2 | | | |
| --- | --- | --- | --- | --- | --- | --- | --- | --- | --- | --- | --- | --- | --- | --- | --- |
|  | Genotype | Elixer | Genius | Leandrus | Ning 0604 |  | Elixer | Genius | Leandrus | Ning 0604 |  | Elixer | Genius | Leandrus | Ning 0604 |
| GY [g] |  |  |  |  |  |  |  |  |  |  |  |  |  |  |  |
| Mean |  | 76.67 | 60.93 | 79.10 | 91.57 |  | 62.60 | 61.48 | 67.35 | 69.00 |  | 88.93 | 63.27 | 81.30 | 67.20 |
| Group |  | ab | b | ab | a |  | a | a | a | a |  | a | b | ab | ab |
| Var |  | 223.76 | 236.74 | 6.03 | 328.32 |  | 169.44 | 96.55 | 48.47 | 175.61 |  | 479.29 | 168.22 | 68.59 | 20.61 |
| SD |  | 14.96 | 15.39 | 2.46 | 18.12 |  | 13.02 | 9.83 | 6.96 | 13.25 |  | 21.89 | 12.97 | 8.28 | 4.54 |
| CoV |  | 0.20 | 0.25 | 0.03 | 0.20 |  | 0.21 | 0.16 | 0.10 | 0.19 |  | 0.25 | 0.21 | 0.10 | 0.07 |
| Min |  | 59.40 | 52.00 | 77.00 | 72.60 |  | 40.70 | 52.00 | 60.20 | 51.60 |  | 70.20 | 49.90 | 71.80 | 63.60 |
| Max |  | 85.70 | 78.70 | 81.80 | 108.70 |  | 74.40 | 76.90 | 79.50 | 87.50 |  | 113.00 | 75.80 | 87.00 | 72.30 |
| TKW [g] |  |  |  |  |  |  |  |  |  |  |  |  |  |  |  |
| Mean |  | 3.76 | 3.66 | 3.75 | 3.89 |  | 3.38 | 3.63 | 3.57 | 3.53 |  | 4.01 | 3.76 | 3.82 | 3.63 |
| Group |  | a | a | a | a |  | a | a | a | a |  | a | a | a | a |
| Var |  | 0.02 | 0.05 | 0.00 | 0.00 |  | 0.06 | 0.09 | 0.04 | 0.05 |  | 0.03 | 0.02 | 0.10 | 0.20 |
| SD |  | 0.14 | 0.21 | 0.06 | 0.04 |  | 0.25 | 0.29 | 0.20 | 0.22 |  | 0.18 | 0.13 | 0.31 | 0.44 |
| CoV |  | 0.04 | 0.06 | 0.01 | 0.01 |  | 0.07 | 0.08 | 0.05 | 0.06 |  | 0.05 | 0.03 | 0.08 | 0.12 |
| Min |  | 3.67 | 3.41 | 3.69 | 3.85 |  | 2.94 | 3.32 | 3.22 | 3.21 |  | 3.88 | 3.64 | 3.51 | 3.32 |
| Max |  | 3.92 | 3.80 | 3.79 | 3.93 |  | 3.63 | 4.01 | 3.80 | 3.81 |  | 4.22 | 3.89 | 4.13 | 4.14 |
| DM [g] |  |  |  |  |  |  |  |  |  |  |  |  |  |  |  |
| Mean |  | 196.93 | 155.12 | 205.94 | 230.58 |  | 175.11 | 197.29 | 187.81 | 205.48 |  | 229.92 | 201.39 | 218.76 | 209.63 |
| Group |  | a | a | a | a |  | a | a | a | a |  | a | a | a | a |
| Var |  | 877.33 | 4716.65 | 136.65 | 1534.21 |  | 951.51 | 915.18 | 231.33 | 627.40 |  | 1672.36 | 110.59 | 340.84 | 94.10 |
| SD |  | 29.62 | 68.68 | 11.69 | 39.17 |  | 30.85 | 30.25 | 15.21 | 25.05 |  | 40.89 | 10.52 | 18.46 | 9.70 |
| CoV |  | 0.15 | 0.44 | 0.06 | 0.17 |  | 0.18 | 0.15 | 0.08 | 0.12 |  | 0.18 | 0.05 | 0.08 | 0.05 |
| Min |  | 162.82 | 81.96 | 192.60 | 186.74 |  | 128.80 | 167.76 | 169.78 | 171.89 |  | 183.62 | 194.30 | 197.45 | 198.71 |
| Max |  | 216.08 | 218.20 | 214.42 | 262.12 |  | 219.08 | 254.00 | 206.55 | 230.89 |  | 261.11 | 213.47 | 229.74 | 217.23 |
| Spike [g] |  |  |  |  |  |  |  |  |  |  |  |  |  |  |  |
| Mean |  | 97.97 | 82.70 | 101.00 | 125.90 |  | 82.43 | 85.62 | 88.93 | 104.12 |  | 112.93 | 88.07 | 105.23 | 104.23 |
| Group |  | ab | b | ab | a |  | a | a | a | a |  | a | b | a | a |
| Var |  | 311.22 | 340.83 | 11.23 | 522.12 |  | 262.53 | 203.36 | 86.69 | 258.97 |  | 588.36 | 126.58 | 112.36 | 1.58 |
| SD |  | 17.64 | 18.46 | 3.35 | 22.85 |  | 16.20 | 14.26 | 9.31 | 16.09 |  | 24.26 | 11.25 | 10.60 | 1.26 |
| CoV |  | 0.18 | 0.22 | 0.03 | 0.18 |  | 0.20 | 0.17 | 0.10 | 0.15 |  | 0.21 | 0.13 | 0.10 | 0.01 |
| Min |  | 77.60 | 71.30 | 97.70 | 101.10 |  | 55.50 | 70.70 | 80.60 | 84.40 |  | 89.00 | 76.90 | 93.00 | 102.90 |
| Max |  | 108.50 | 104.00 | 104.40 | 146.10 |  | 100.00 | 109.70 | 103.90 | 124.00 |  | 137.50 | 99.40 | 111.70 | 105.40 |
| Straw [g] |  |  |  |  |  |  |  |  |  |  |  |  |  |  |  |
| Mean |  | 98.97 | 72.42 | 104.94 | 104.68 |  | 92.67 | 111.67 | 98.87 | 101.36 |  | 116.99 | 113.32 | 113.53 | 105.40 |
| Group |  | a | b | a | a |  | b | a | ab | ab |  | a | a | a | a |
| Var |  | 144.83 | 79.52 | 75.51 | 75.33 |  | 239.59 | 281.22 | 56.37 | 15.09 |  | 396.04 | 427.53 | 61.86 | 71.69 |
| SD |  | 12.03 | 54.58 | 8.69 | 16.59 |  | 15.48 | 16.77 | 7.51 | 10.73 |  | 19.90 | 20.68 | 7.86 | 8.47 |
| CoV |  | 0.12 | 0.75 | 0.08 | 0.16 |  | 0.17 | 0.15 | 0.08 | 0.11 |  | 0.17 | 0.18 | 0.07 | 0.08 |
| Min |  | 85.22 | 10.66 | 94.90 | 85.64 |  | 73.30 | 97.06 | 87.38 | 85.29 |  | 94.62 | 96.99 | 104.45 | 95.81 |
| Max |  | 107.58 | 114.20 | 110.02 | 116.02 |  | 119.08 | 144.30 | 109.95 | 113.79 |  | 132.74 | 136.57 | 118.11 | 111.83 |
| RDM [g] |  |  |  |  |  |  |  |  |  |  |  |  |  |  |  |
| Mean |  | 166.83 | 102.50 | 86.63 | 100.19 |  | 145.43 | 113.28 | 113.72 | 80.31 |  | 192.33 | 108.83 | 108.33 | 108.16 |
| Group |  | a | b | b | b |  | a | ab | ab | b |  | a | b | b | b |
| Var |  | 1641.86 | 95.93 | 485.33 | 214.14 |  | 210.21 | 437.21 | 173.37 | 342.64 |  | 2746.30 | 1371.09 | 199.09 | 850.41 |
| SD |  | 40.52 | 38.95 | 22.03 | 14.63 |  | 14.50 | 20.91 | 13.17 | 18.51 |  | 52.41 | 37.03 | 14.11 | 29.16 |
| CoV |  | 0.24 | 0.38 | 0.25 | 0.15 |  | 0.10 | 0.18 | 0.12 | 0.23 |  | 0.27 | 0.34 | 0.13 | 0.27 |
| Min |  | 125.60 | 67.40 | 65.30 | 85.38 |  | 118.50 | 93.10 | 98.10 | 60.13 |  | 143.80 | 85.10 | 99.40 | 86.77 |
| Max |  | 206.60 | 144.40 | 109.30 | 114.64 |  | 156.20 | 148.60 | 133.00 | 106.86 |  | 247.90 | 151.50 | 124.60 | 141.38 |
