# Supplementary material for: Effect of a QTL on wheat chromosome 5B associated with enhanced root dry mass on transpiration and nitrogen uptake under contrasting drought scenarios in wheat
Source: BMC Plant Biol. 2024 Feb 2;24:83. doi: 10.1186/s12870-024-04756-8 (PMC10835935; doi:10.1186/s12870-024-04756-8)
Supplement: Supplementary file 3 — Additional file 3. Descriptive statistics for collected harvest data in experiment 2 [file 12870_2024_4756_MOESM3_ESM.docx]

Additional file 3: Descriptive statistics for collected harvest data in experiment 2

| Trait | Genotype | Elixer | | | |  | Genius | | | |  | Leandrus | | | |
| --- | --- | --- | --- | --- | --- | --- | --- | --- | --- | --- | --- | --- | --- | --- | --- |
|  | Treatment | Well-Watered | | Drought | |  | Well-Watered | | Drought | |  | Well-Watered | | Drought | |
|  | Background | PAR | NIL | PAR | NIL |  | PAR | NIL | PAR | NIL |  | PAR | NIL | PAR | NIL |
| FW [g] |  |  |  |  |  |  |  |  |  |  |  |  |  |  |  |
| Mean |  | 360.88 | 338.56 | 242.78 | 241.44 |  | 348.81 | 370.25 | 223.77 | 202.97 |  | 319.48 | 331.05 | 210.41 | 177.38 |
| Group |  | a | a | a | a |  | a | a | a | a |  | a | a | a | a |
| Var |  | 538.87 | 436.56 | 2027.6 | 1119.8 |  | 119.25 | 385.5 | 559.75 | 694.64 |  | 341.27 | 664.7 | 1156.5 | 4591.6 |
| SD |  | 23.21 | 20.89 | 45.03 | 33.46 |  | 10.92 | 19.63 | 23.66 | 26.36 |  | 18.47 | 25.78 | 34.01 | 67.76 |
| CoV |  | 0.06 | 0.06 | 0.19 | 0.14 |  | 0.03 | 0.05 | 0.11 | 0.13 |  | 0.06 | 0.08 | 0.16 | 0.38 |
| Min |  | 334.69 | 318.33 | 191.14 | 202.82 |  | 337.84 | 355.05 | 208.08 | 172.55 |  | 299.86 | 306.65 | 172.31 | 103.03 |
| Max |  | 378.91 | 360.06 | 273.85 | 261.93 |  | 359.68 | 392.42 | 250.98 | 218.96 |  | 336.54 | 358.02 | 237.70 | 235.67 |
| DM [g] |  |  |  |  |  |  |  |  |  |  |  |  |  |  |  |
| Mean |  | 67.95 | 60.82 | 49.64 | 49.13 |  | 64.88 | 66.56 | 46.54 | 43.13 |  | 64.87 | 61.84 | 46.20 | 38.37 |
| Group |  | a | a | a | a |  | a | a | a | a |  | a | a | a | a |
| Var |  | 26.084 | 11.197 | 142.17 | 59.738 |  | 21.249 | 56.886 | 48.761 | 10.413 |  | 0.4837 | 66.96 | 82.308 | 151.48 |
| SD |  | 5.11 | 3.35 | 11.92 | 7.73 |  | 4.61 | 7.54 | 6.98 | 3.23 |  | 0.70 | 8.18 | 9.07 | 12.31 |
| CoV |  | 0.08 | 0.06 | 0.24 | 0.16 |  | 0.07 | 0.11 | 0.15 | 0.07 |  | 0.01 | 0.13 | 0.20 | 0.32 |
| Min |  | 62.69 | 58.83 | 35.98 | 40.28 |  | 61.03 | 57.96 | 40.81 | 39.84 |  | 64.34 | 52.56 | 35.76 | 24.69 |
| Max |  | 72.89 | 64.68 | 57.98 | 54.57 |  | 69.99 | 72.05 | 54.32 | 46.29 |  | 65.66 | 68.02 | 52.17 | 48.55 |
| RDM [g] |  |  |  |  |  |  |  |  |  |  |  |  |  |  |  |
| Mean |  | 14.62 | 10.56 | 17.33 | 18.61 |  | 6.04 | 15.41 | 14.77 | 27.30 |  | 13.55 | 18.16 | 14.08 | 19.06 |
| Group |  | a | a | a | a |  | a | a | a | a |  | a | a | a | a |
| Var |  | 71.824 | 2.3377 | 153.65 | 83.284 |  | 3.926 | 34.886 | 21.172 | 37.278 |  | 41.604 | 6.7609 | 45.816 | 209.64 |
| SD |  | 8.47 | 1.53 | 12.40 | 9.13 |  | 1.98 | 5.91 | 4.60 | 6.11 |  | 6.45 | 2.60 | 6.77 | 14.48 |
| CoV |  | 0.58 | 0.14 | 0.72 | 0.49 |  | 0.33 | 0.38 | 0.31 | 0.22 |  | 0.48 | 0.14 | 0.48 | 0.76 |
| Min |  | 6.50 | 8.83 | 5.37 | 8.22 |  | 4.02 | 10.65 | 10.93 | 21.61 |  | 6.15 | 15.77 | 7.91 | 4.39 |
| Max |  | 23.41 | 11.73 | 30.12 | 25.34 |  | 7.98 | 22.02 | 19.87 | 33.75 |  | 17.96 | 20.93 | 21.32 | 33.34 |
