# Supplementary material for: Effect of a QTL on wheat chromosome 5B associated with enhanced root dry mass on transpiration and nitrogen uptake under contrasting drought scenarios in wheat
Source: BMC Plant Biol. 2024 Feb 2;24:83. doi: 10.1186/s12870-024-04756-8 (PMC10835935; doi:10.1186/s12870-024-04756-8)
Supplement: Supplementary file 4 — Additional file 4. Digital growth parameters recorded in experiment 1 using the PlantEye F500® multi-spectral 3D laser scanner [file 12870_2024_4756_MOESM4_ESM.docx]

Additional file 4: Digital growth parameters recorded in experiment 1 using the PlantEye F500^®^ multi-spectral 3D laser scanner

| Trait | DAT | Treatment | Well-Watered | | | |  | Drought Scenario 1 | | | |  | Drought Scenario 2 | | | |
| --- | --- | --- | --- | --- | --- | --- | --- | --- | --- | --- | --- | --- | --- | --- | --- | --- |
|  |  | Genotype | Elixer | Genius | Leandrus | Ning 0604 |  | Elixer | Genius | Leandrus | Ning 0604 |  | Elixer | Genius | Leandrus | Ning 0604 |
| Digital Biomass [dm³] | -42 |  | NA | NA | 0.02 | NA |  | NA | NA | 0.02 | NA |  | NA | NA | 0.02 | NA |
|  |  |  | NA | NA | b | NA |  | NA | NA | c | NA |  | NA | NA | b | NA |
|  |  |  | NA | NA | 0.00 | NA |  | NA | NA | 0.00 | NA |  | NA | NA | 0.00 | NA |
|  | -35 |  | 0.03 | 0.03 | 0.05 | NA |  | 0.03 | 0.03 | 0.05 | NA |  | 0.03 | 0.04 | 0.05 | NA |
|  |  |  | b | b | b | NA |  | bc | b | b | NA |  | b | b | b | NA |
|  |  |  | 0.00 | 0.00 | 0.00 | NA |  | 0.01 | 0.01 | 0.01 | NA |  | 0.01 | 0.00 | 0.00 | NA |
|  | -28 |  | 0.07 | 0.06 | 0.09 | NA |  | 0.06 | 0.08 | 0.09 | NA |  | 0.07 | 0.07 | 0.09 | NA |
|  |  |  | b | b | b | NA |  | b | b | c | NA |  | b | b | b | NA |
|  |  |  | 0.01 | 0.00 | 0.01 | NA |  | 0.01 | 0.02 | 0.01 | NA |  | 0.02 | 0.00 | 0.01 | NA |
|  | -21 |  | 0.12 | 0.10 | 0.12 | NA |  | 0.11 | 0.14 | 0.13 | NA |  | 0.12 | 0.12 | 0.12 | NA |
|  |  |  | b | b | c | NA |  | bc | b | c | NA |  | b | b | c | NA |
|  |  |  | 0.02 | 0.00 | 0.01 | NA |  | 0.01 | 0.02 | 0.01 | NA |  | 0.02 | 0.02 | 0.00 | NA |
|  | -14 |  | 0.17 | 0.15 | 0.16 | 0.17 |  | 0.16 | 0.19 | 0.16 | 0.15 |  | 0.17 | 0.17 | 0.16 | 0.16 |
|  |  |  | b | bc | c | a |  | b | b | c | a |  | b | b | c | a |
|  |  |  | 0.02 | 0.01 | 0.01 | 0.01 |  | 0.02 | 0.03 | 0.02 | 0.02 |  | 0.03 | 0.03 | 0.01 | 0.03 |
|  | -7 |  | 0.21 | 0.18 | 0.21 | 0.30 |  | 0.20 | 0.22 | 0.20 | 0.24 |  | 0.22 | 0.21 | 0.21 | 0.27 |
|  |  |  | b | bc | b | a |  | b | b | c | a |  | b | b | b | a |
|  |  |  | 0.03 | 0.01 | 0.01 | 0.04 |  | 0.02 | 0.03 | 0.04 | 0.05 |  | 0.03 | 0.03 | 0.01 | 0.01 |
|  | HD |  | 0.24 | 0.22 | 0.26 | 0.37 |  | 0.24 | 0.28 | 0.24 | 0.34 |  | 0.27 | 0.26 | 0.24 | 0.36 |
|  |  |  | b | b | b | a |  | bc | b | c | a |  | b | b | b | a |
|  |  |  | 0.04 | 0.03 | 0.03 | 0.04 |  | 0.04 | 0.05 | 0.03 | 0.06 |  | 0.03 | 0.05 | 0.01 | 0.02 |
|  | 7 |  | 0.25 | 0.23 | 0.24 | 0.36 |  | 0.25 | 0.29 | 0.22 | 0.36 |  | 0.29 | 0.29 | 0.25 | 0.35 |
|  |  |  | b | b | a | a |  | bc | b | b | a |  | b | b | a | a |
|  |  |  | 0.04 | 0.02 | 0.01 | 0.03 |  | 0.03 | 0.06 | 0.03 | 0.03 |  | 0.02 | 0.05 | 0.01 | 0.01 |
|  | 14 |  | 0.25 | 0.22 | 0.24 | 0.34 |  | 0.24 | 0.28 | 0.20 | 0.33 |  | 0.30 | 0.28 | 0.22 | 0.33 |
|  |  |  | a | a | a | a |  | ab | a | b | a |  | a | a | a | a |
|  |  |  | 0.04 | 0.01 | 0.02 | 0.04 |  | 0.04 | 0.05 | 0.03 | 0.04 |  | 0.04 | 0.07 | 0.03 | 0.01 |
|  | 21 |  | 0.23 | 0.20 | 0.21 | 0.41 |  | 0.22 | 0.25 | 0.20 | 0.39 |  | 0.27 | 0.24 | 0.21 | 0.38 |
|  |  |  | a | a | a | a |  | ab | a | b | a |  | a | a | a | a |
|  |  |  | 0.04 | 0.02 | 0.02 | 0.05 |  | 0.04 | 0.05 | 0.02 | 0.06 |  | 0.02 | 0.05 | 0.03 | 0.05 |
|  | 28 |  | 0.22 | 0.18 | 0.21 | 0.42 |  | 0.19 | 0.24 | NA | 0.39 |  | 0.23 | 0.21 | 0.20 | 0.38 |
|  |  |  | a | a | a | a |  | b | a | NA | a |  | a | a | a | a |
|  |  |  | 0.02 | 0.02 | 0.01 | 0.06 |  | 0.02 | 0.04 | NA | NA |  | 0.01 | 0.04 | 0.03 | 0.04 |
|  | 35 |  | 0.20 | 0.18 | NA | NA |  | 0.19 | 0.23 | NA | NA |  | 0.22 | 0.21 | NA | NA |
|  |  |  | a | a | NA | NA |  | a | a | NA | NA |  | a | a | NA | NA |
|  |  |  | 0.03 | 0.02 | NA | NA |  | 0.03 | 0.05 | NA | NA |  | 0.01 | 0.02 | NA | NA |
| Digital Plant Height [cm] | -42 |  | NA | NA | 24.15 | NA |  | NA | NA | 23.35 | NA |  | NA | NA | 25.45 | NA |
|  |  |  | NA | NA | c | NA |  | NA | NA | d | NA |  | NA | NA | c | NA |
|  |  |  | NA | NA | 0.37 | NA |  | NA | NA | 1.99 | NA |  | NA | NA | 0.73 | NA |
|  | -35 |  | 28.48 | 29.29 | 34.30 | NA |  | 27.66 | 30.56 | 33.92 | NA |  | 30.09 | 31.62 | 35.05 | NA |
|  |  |  | b | b | c | NA |  | c | b | c | NA |  | bc | b | c | NA |
|  |  |  | 1.76 | 1.13 | 0.43 | NA |  | 2.33 | 1.28 | 1.28 | NA |  | 2.34 | 0.71 | 0.58 | NA |
|  | -28 |  | 37.86 | 37.37 | 44.35 | NA |  | 37.83 | 40.02 | 44.55 | NA |  | 39.61 | 39.70 | 45.31 | NA |
|  |  |  | b | b | c | NA |  | bc | b | c | NA |  | bc | b | c | NA |
|  |  |  | 1.77 | 1.07 | 2.27 | NA |  | 2.30 | 2.36 | 2.36 | NA |  | 2.66 | 2.02 | 2.53 | NA |
|  | -21 |  | 49.26 | 47.79 | 51.70 | NA |  | 49.20 | 52.77 | 52.15 | NA |  | 50.55 | 51.42 | 52.14 | NA |
|  |  |  | b | bc | c | NA |  | bc | b | d | NA |  | b | b | c | NA |
|  |  |  | 3.10 | 1.75 | 2.77 | NA |  | 2.07 | 2.41 | 1.85 | NA |  | 2.66 | 3.08 | 2.09 | NA |
|  | -14 |  | 57.73 | 56.04 | 58.18 | 59.17 |  | 57.30 | 61.28 | 57.97 | 57.32 |  | 58.66 | 60.07 | 58.29 | 58.52 |
|  |  |  | b | bc | c | a |  | c | b | c | a |  | b | b | c | a |
|  |  |  | 3.65 | 1.75 | 2.21 | 1.43 |  | 2.30 | 2.58 | 2.70 | 3.44 |  | 3.37 | 3.08 | 2.14 | 4.54 |
|  | -7 |  | 65.49 | 63.56 | 69.91 | 74.78 |  | 65.12 | 68.41 | 68.88 | 69.88 |  | 67.38 | 67.66 | 69.23 | 74.67 |
|  |  |  | b | b | b | a |  | b | b | c | a |  | b | b | c | a |
|  |  |  | 3.22 | 1.89 | 1.38 | 0.89 |  | 2.64 | 3.60 | 4.10 | 8.02 |  | 3.02 | 4.01 | 1.89 | 3.51 |
|  | HD |  | 71.64 | 71.74 | 75.78 | 88.66 |  | 72.17 | 78.42 | 74.55 | 85.05 |  | 75.55 | 77.89 | 74.42 | 88.74 |
|  |  |  | b | b | b | a |  | bc | b | b | a |  | bc | b | b | a |
|  |  |  | 4.94 | 5.26 | 1.49 | 2.17 |  | 4.78 | 6.08 | 4.74 | 8.07 |  | 2.22 | 5.81 | 1.07 | 1.14 |
|  | 7 |  | 74.05 | 73.81 | 76.75 | 89.02 |  | 74.91 | 80.31 | 73.28 | 88.13 |  | 79.77 | 80.09 | 76.59 | 88.69 |
|  |  |  | b | b | a | a |  | b | b | b | a |  | b | b | a | a |
|  |  |  | 4.70 | 3.52 | 1.49 | 2.06 |  | 5.09 | 6.32 | 4.63 | 3.33 |  | 0.83 | 6.32 | 1.35 | 1.26 |
|  | 14 |  | 74.68 | 74.19 | 76.01 | 88.59 |  | 75.12 | 80.28 | 73.16 | 88.16 |  | 79.47 | 80.31 | 73.58 | 88.19 |
|  |  |  | a | a | a | a |  | ab | a | a | a |  | a | a | a | a |
|  |  |  | 4.78 | 3.85 | 1.32 | 2.48 |  | 5.41 | 6.37 | 4.45 | 3.33 |  | 0.72 | 6.13 | 4.96 | 0.37 |
|  | 21 |  | 73.61 | 72.10 | 75.83 | 111.52 |  | 73.81 | 79.51 | 72.54 | 107.73 |  | 78.36 | 79.27 | 73.27 | 107.82 |
|  |  |  | a | a | a | a |  | a | a | b | a |  | a | a | a | a |
|  |  |  | 4.67 | 4.55 | 1.33 | 3.51 |  | 5.28 | 6.54 | 4.00 | 11.70 |  | 0.53 | 5.89 | 5.17 | 6.51 |
|  | 28 |  | 72.83 | 71.16 | 75.84 | 111.61 |  | 72.48 | 79.18 | NA | 113.63 |  | 77.53 | 77.48 | 72.97 | 108.16 |
|  |  |  | a | a | a | a |  | b | a | NA | a |  | a | a | a | a |
|  |  |  | 4.93 | 5.37 | 1.38 | 3.50 |  | 4.04 | 6.64 | NA | NA |  | 0.20 | 4.37 | 6.09 | 5.96 |
|  | 35 |  | 72.78 | 71.69 | NA | NA |  | 71.87 | 78.78 | NA | NA |  | 76.58 | 77.67 | NA | NA |
|  |  |  | a | a | NA | NA |  | a | a | NA | NA |  | a | a | NA | NA |
|  |  |  | 4.87 | 5.58 | NA | NA |  | 4.13 | 6.61 | NA | NA |  | 0.53 | 3.85 | NA | NA |
| NDVI | -42 |  | NA | NA | 0.55 | NA |  | NA | NA | 0.55 | NA |  | NA | NA | 0.56 | NA |
|  |  |  | NA | NA | ab | NA |  | NA | NA | b | NA |  | NA | NA | a | NA |
|  |  |  | NA | NA | 0.01 | NA |  | NA | NA | 0.01 | NA |  | NA | NA | 0.01 | NA |
|  | -35 |  | 0.57 | 0.56 | 0.55 | NA |  | 0.56 | 0.56 | 0.54 | NA |  | 0.57 | 0.56 | 0.55 | NA |
|  |  |  | a | a | a | NA |  | ab | a | a | NA |  | a | a | a | NA |
|  |  |  | 0.02 | 0.01 | 0.01 | NA |  | 0.02 | 0.00 | 0.01 | NA |  | 0.02 | 0.01 | 0.02 | NA |
|  | -28 |  | 0.56 | 0.55 | 0.47 | NA |  | 0.55 | 0.55 | 0.48 | NA |  | 0.56 | 0.55 | 0.48 | NA |
|  |  |  | a | a | a | NA |  | a | a | a | NA |  | a | a | a | NA |
|  |  |  | 0.01 | 0.00 | 0.01 | NA |  | 0.02 | 0.02 | 0.02 | NA |  | 0.01 | 0.01 | 0.04 | NA |
|  | -21 |  | 0.50 | 0.50 | 0.42 | NA |  | 0.49 | 0.50 | 0.43 | NA |  | 0.51 | 0.48 | 0.44 | NA |
|  |  |  | a | a | a | NA |  | a | a | a | NA |  | a | a | a | NA |
|  |  |  | 0.02 | 0.02 | 0.01 | NA |  | 0.02 | 0.03 | 0.02 | NA |  | 0.00 | 0.02 | 0.04 | NA |
|  | -14 |  | 0.45 | 0.44 | 0.37 | 0.51 |  | 0.44 | 0.44 | 0.38 | 0.53 |  | 0.47 | 0.43 | 0.38 | 0.51 |
|  |  |  | a | a | a | b |  | a | a | a | c |  | a | a | a | b |
|  |  |  | 0.03 | 0.03 | 0.01 | 0.03 |  | 0.02 | 0.04 | 0.02 | 0.02 |  | 0.01 | 0.02 | 0.04 | 0.01 |
|  | -7 |  | 0.39 | 0.38 | 0.31 | 0.43 |  | 0.39 | 0.38 | 0.32 | 0.46 |  | 0.40 | 0.37 | 0.32 | 0.44 |
|  |  |  | a | a | a | b |  | a | a | a | b |  | a | a | a | b |
|  |  |  | 0.03 | 0.03 | 0.00 | 0.05 |  | 0.02 | 0.04 | 0.02 | 0.04 |  | 0.02 | 0.01 | 0.02 | 0.02 |
|  | HD |  | 0.33 | 0.33 | 0.26 | 0.36 |  | 0.34 | 0.32 | 0.26 | 0.40 |  | 0.34 | 0.33 | 0.27 | 0.38 |
|  |  |  | a | a | a | b |  | a | a | b | b |  | a | a | b | b |
|  |  |  | 0.01 | 0.02 | 0.00 | 0.03 |  | 0.02 | 0.03 | 0.02 | 0.04 |  | 0.02 | 0.02 | 0.01 | 0.03 |
|  | 7 |  | 0.30 | 0.29 | 0.26 | 0.35 |  | 0.29 | 0.27 | 0.22 | 0.36 |  | 0.33 | 0.31 | 0.27 | 0.36 |
|  |  |  | a | a | a | b |  | a | ab | c | b |  | a | ab | a | b |
|  |  |  | 0.01 | 0.04 | 0.01 | 0.03 |  | 0.02 | 0.02 | 0.01 | 0.04 |  | 0.03 | 0.02 | 0.01 | 0.02 |
|  | 14 |  | 0.27 | 0.26 | 0.24 | 0.28 |  | 0.27 | 0.24 | 0.16 | 0.29 |  | 0.31 | 0.28 | 0.24 | 0.30 |
|  |  |  | a | a | a | b |  | a | b | b | b |  | a | a | a | b |
|  |  |  | 0.01 | 0.04 | 0.01 | 0.03 |  | 0.02 | 0.02 | 0.01 | 0.03 |  | 0.04 | 0.01 | 0.00 | 0.02 |
|  | 21 |  | 0.21 | 0.20 | 0.18 | 0.20 |  | 0.19 | 0.18 | 0.13 | 0.22 |  | 0.24 | 0.20 | 0.17 | 0.21 |
|  |  |  | b | b | a | b |  | a | a | a | b |  | a | a | a | b |
|  |  |  | 0.01 | 0.02 | 0.01 | 0.02 |  | 0.02 | 0.01 | 0.01 | 0.03 |  | 0.04 | 0.01 | 0.00 | 0.02 |
|  | 28 |  | 0.14 | 0.13 | 0.14 | 0.16 |  | 0.13 | 0.12 | NA | 0.21 |  | 0.16 | 0.12 | 0.13 | 0.17 |
|  |  |  | b | b | a | b |  | a | a | NA | c |  | ab | b | ab | c |
|  |  |  | 0.00 | 0.02 | 0.01 | 0.01 |  | 0.01 | 0.01 | NA | NA |  | 0.03 | 0.01 | 0.01 | 0.02 |
|  | 35 |  | 0.11 | 0.12 | NA | NA |  | 0.11 | 0.11 | NA | NA |  | 0.13 | 0.11 | NA | NA |
|  |  |  | b | b | NA | NA |  | a | a | NA | NA |  | a | b | NA | NA |
|  |  |  | 0.00 | 0.01 | NA | NA |  | 0.01 | 0.01 | NA | NA |  | 0.01 | 0.01 | NA | NA |
