# Supplementary material for: Effect of a QTL on wheat chromosome 5B associated with enhanced root dry mass on transpiration and nitrogen uptake under contrasting drought scenarios in wheat
Source: BMC Plant Biol. 2024 Feb 2;24:83. doi: 10.1186/s12870-024-04756-8 (PMC10835935; doi:10.1186/s12870-024-04756-8)
Supplement: Supplementary file 5 — Additional file 5. Descriptive statistics for transpiration data as well as physiological growth parameters recorded in experiment 2 from -7 DAT until harvest [file 12870_2024_4756_MOESM5_ESM.docx]

Additional file 5: Descriptive statistics for transpiration data as well as physiological growth parameters recorded in experiment 2 from -7 DAT until harvest

| Trait | DAT | Genotype | Elixer | | | |  | Genius | | | |  | Leandrus | | | |
| --- | --- | --- | --- | --- | --- | --- | --- | --- | --- | --- | --- | --- | --- | --- | --- | --- |
|  |  | Treatment | Well-Watered | | Drought | |  | Well-Watered | | Drought | |  | Well-Watered | | Drought | |
|  |  | Background | PAR | NIL | PAR | NIL |  | PAR | NIL | PAR | NIL |  | PAR | NIL | PAR | NIL |
| Cumulative Transpiration [L] | -7 | Mean | 0.76 | 0.73 | 0.66 | 0.78 |  | 0.77 | 0.70 | 0.94 | 1.04 |  | 0.77 | 0.66 | 0.67 | 0.62 |
|  |  | Group | a | a | a | a |  | a | a | a | a |  | a | a | a | a |
|  |  | SD | 0.10 | 0.01 | 0.22 | 0.10 |  | 0.17 | 0.18 | 0.30 | 0.61 |  | 0.06 | 0.18 | 0.15 | 0.18 |
|  | 0 | Mean | 1.53 | 1.45 | 1.09 | 1.24 |  | 1.52 | 1.37 | 1.40 | 1.49 |  | 1.57 | 1.32 | 1.11 | 0.96 |
|  |  | Group | a | a | a | a |  | a | a | a | a |  | a | a | a | a |
|  |  | SD | 0.15 | 0.04 | 0.29 | 0.13 |  | 0.29 | 0.37 | 0.29 | 0.68 |  | 0.13 | 0.34 | 0.21 | 0.23 |
|  | 7 | Mean | 2.75 | 2.55 | 1.43 | 1.48 |  | 2.66 | 2.42 | 1.62 | 1.69 |  | 2.86 | 2.30 | 1.40 | 1.07 |
|  |  | Group | a | a | a | a |  | a | a | a | a |  | a | a | a | a |
|  |  | SD | 0.23 | 0.09 | 0.38 | 0.21 |  | 0.45 | 0.62 | 0.31 | 0.72 |  | 0.22 | 0.53 | 0.29 | 0.29 |
|  | 14 | Mean | 4.35 | 3.90 | 2.20 | 2.25 |  | 4.14 | 3.73 | 2.27 | 2.28 |  | 4.49 | 3.46 | 2.14 | 1.36 |
|  |  | Group | a | a | a | a |  | a | a | a | a |  | a | a | a | a |
|  |  | SD | 0.37 | 0.16 | 0.69 | 0.37 |  | 0.73 | 0.97 | 0.41 | 0.82 |  | 0.37 | 0.84 | 0.54 | 0.53 |
|  | 21 | Mean | 6.72 | 5.88 | 3.62 | 3.62 |  | 6.34 | 5.66 | 3.49 | 3.32 |  | 6.83 | 5.11 | 3.43 | 2.02 |
|  |  | Group | a | b | a | a |  | a | a | a | a |  | a | a | a | a |
|  |  | SD | 0.58 | 0.30 | 1.13 | 0.51 |  | 1.07 | 1.48 | 0.58 | 0.96 |  | 0.67 | 1.29 | 0.96 | 0.90 |
|  | 28 | Mean | 9.71 | 8.40 | 5.55 | 5.44 |  | 9.05 | 8.23 | 5.14 | 4.77 |  | 9.71 | 7.27 | 5.19 | 3.05 |
|  |  | Group | a | b | a | a |  | a | a | a | a |  | a | a | a | a |
|  |  | SD | 0.86 | 0.46 | 1.65 | 0.77 |  | 1.53 | 2.28 | 0.90 | 1.12 |  | 0.93 | 1.93 | 1.55 | 1.47 |
|  | 35 | Mean | 13.31 | 11.51 | 7.96 | 7.80 |  | 12.42 | 11.57 | 7.21 | 6.68 |  | 13.29 | 10.16 | 7.38 | 4.48 |
|  |  | Group | a | b | a | a |  | a | a | a | a |  | a | a | a | a |
|  |  | SD | 1.26 | 0.55 | 2.25 | 1.23 |  | 2.02 | 3.25 | 1.29 | 1.31 |  | 1.25 | 2.72 | 2.22 | 2.23 |
|  | 42 | Mean | 17.22 | 14.77 | 10.70 | 10.50 |  | 16.00 | 15.40 | 9.62 | 9.01 |  | 17.35 | 13.58 | 9.79 | 6.21 |
|  |  | Group | a | b | a | a |  | a | a | a | a |  | a | a | a | a |
|  |  | SD | 1.68 | 0.55 | 3.11 | 1.93 |  | 2.55 | 4.32 | 1.87 | 1.45 |  | 1.49 | 3.67 | 3.06 | 3.14 |
|  | 56 | Mean | 26.21 | 22.76 | 17.41 | 17.19 |  | 24.09 | 24.33 | 15.82 | 15.04 |  | 26.43 | 22.37 | 15.76 | 11.22 |
|  |  | Group | a | b | a | a |  | a | a | a | a |  | a | a | a | a |
|  |  | SD | 2.47 | 0.50 | 4.84 | 3.21 |  | 2.92 | 5.55 | 3.15 | 1.74 |  | 1.43 | 4.88 | 4.76 | 5.39 |
|  | Harvest | Mean | 30.79 | 27.14 | 21.43 | 21.13 |  | 28.19 | 29.26 | 19.41 | 18.70 |  | 30.94 | 27.34 | 19.21 | 14.32 |
|  |  | Group | a | b | a | a |  | a | a | a | a |  | a | a | a | a |
|  |  | SD | 2.75 | 0.31 | 5.28 | 3.64 |  | 2.66 | 5.23 | 3.38 | 1.89 |  | 1.20 | 4.78 | 5.39 | 6.46 |
|  |  |  |  |  |  |  |  |  |  |  |  |  |  |  |  |  |
| Dayli Transpiration [ml] | -7 | Mean | 171.70 | 166.80 | 116.70 | 143.10 |  | 175.10 | 162.86 | 133.70 | 142.86 |  | 180.93 | 153.80 | 146.26 | 119.80 |
|  |  | Group | a | a | a | a |  | a | a | a | a |  | a | a | a | a |
|  |  | SD | 6.20 | 2.50 | 55.34 | 13.99 |  | 22.91 | 27.10 | 25.76 | 17.71 |  | 12.66 | 30.61 | 16.20 | 18.07 |
|  | 0 | Mean | 144.38 | 128.05 | 45.58 | 43.82 |  | 133.52 | 123.02 | 44.88 | 42.88 |  | 151.78 | 118.55 | 50.68 | 28.88 |
|  |  | Group | a | b | a | a |  | a | a | a | a |  | a | a | a | b |
|  |  | SD | 10.17 | 7.64 | 10.04 | 9.38 |  | 21.53 | 33.35 | 9.84 | 15.37 |  | 8.21 | 26.11 | 6.44 | 4.22 |
|  | 7 | Mean | 181.37 | 152.67 | 63.00 | 47.83 |  | 163.17 | 148.90 | 42.27 | 39.13 |  | 189.03 | 137.73 | 61.30 | 25.52 |
|  |  | Group | a | b | a | a |  | a | a | a | a |  | a | b | a | a |
|  |  | SD | 13.33 | 9.54 | 42.33 | 28.03 |  | 35.33 | 36.37 | 27.81 | 14.40 |  | 18.12 | 31.15 | 36.21 | 5.87 |
|  | 14 | Mean | 291.68 | 250.05 | 157.95 | 159.95 |  | 279.45 | 240.08 | 140.92 | 123.58 |  | 295.82 | 209.42 | 146.55 | 92.98 |
|  |  | Group | a | b | a | a |  | a | a | a | a |  | a | b | a | a |
|  |  | SD | 18.18 | 18.13 | 58.77 | 12.08 |  | 46.61 | 62.22 | 26.94 | 24.64 |  | 31.96 | 58.73 | 45.01 | 0.57 |
|  | 21 | Mean | 373.80 | 309.27 | 232.10 | 221.67 |  | 335.77 | 303.50 | 194.73 | 171.83 |  | 363.37 | 257.33 | 208.97 | 108.70 |
|  |  | Group | a | b | a | a |  | a | a | a | a |  | a | a | a | a |
|  |  | SD | 33.74 | 27.20 | 71.03 | 21.56 |  | 56.98 | 87.75 | 39.89 | 29.72 |  | 50.32 | 74.36 | 76.22 | 59.22 |
|  | 28 | Mean | 468.53 | 407.17 | 305.03 | 301.20 |  | 440.27 | 421.53 | 262.33 | 236.37 |  | 462.80 | 355.47 | 284.17 | 174.07 |
|  |  | Group | a | a | a | a |  | a | a | a | a |  | a | a | a | a |
|  |  | SD | 43.75 | 27.41 | 80.54 | 41.39 |  | 71.95 | 131.56 | 53.62 | 42.84 |  | 39.95 | 103.51 | 89.73 | 104.46 |
|  | 35 | Mean | 487.23 | 407.33 | 343.90 | 328.13 |  | 454.20 | 476.23 | 296.43 | 280.83 |  | 515.17 | 417.83 | 311.27 | 215.67 |
|  |  | Group | a | a | a | a |  | a | a | a | a |  | a | a | a | a |
|  |  | SD | 66.81 | 6.92 | 110.97 | 90.51 |  | 90.15 | 152.28 | 69.67 | 34.84 |  | 61.59 | 125.07 | 111.52 | 112.78 |
|  | 42 | Mean | 691.85 | 592.12 | 462.05 | 479.28 |  | 639.22 | 672.65 | 418.42 | 410.65 |  | 712.68 | 616.92 | 412.58 | 299.68 |
|  |  | Group | a | b | a | a |  | a | a | a | a |  | a | a | a | a |
|  |  | SD | 62.88 | 37.72 | 135.02 | 90.73 |  | 38.81 | 159.78 | 93.73 | 42.03 |  | 20.07 | 132.40 | 124.12 | 153.97 |
|  | 56 | Mean | 630.45 | 586.78 | 532.15 | 526.62 |  | 583.38 | 681.62 | 493.22 | 506.38 |  | 650.05 | 696.95 | 478.38 | 428.62 |
|  |  | Group | a | a | a | a |  | b | a | a | a |  | a | a | a | a |
|  |  | SD | 53.21 | 18.54 | 93.95 | 70.60 |  | 16.49 | 40.22 | 56.15 | 33.48 |  | 27.95 | 46.65 | 109.95 | 172.58 |
|  | Harvest | Mean | 669.22 | 643.88 | 607.25 | 586.72 |  | 605.55 | 735.18 | 538.62 | 553.32 |  | 664.48 | 748.38 | 518.62 | 478.35 |
|  |  | Group | a | a | a | a |  | b | a | a | a |  | a | a | a | a |
|  |  | SD | 67.49 | 58.85 | 45.02 | 66.58 |  | 46.71 | 82.67 | 41.36 | 26.21 |  | 53.40 | 47.53 | 72.29 | 166.34 |
|  |  |  |  |  |  |  |  |  |  |  |  |  |  |  |  |  |
| Leaf Area [cm²] | -7 | Mean | 4.44 | 4.01 | 5.56 | 4.05 |  | 5.15 | 3.65 | 4.57 | 3.68 |  | 5.80 | 3.26 | 6.19 | 3.28 |
|  |  | Group | a | a | a | b |  | a | b | a | a |  | a | b | a | b |
|  |  | SD | 0.53 | 0.75 | 0.62 | 0.52 |  | 0.25 | 0.29 | 0.68 | 0.14 |  | 0.61 | 0.89 | 0.16 | 0.43 |
|  | 0 | Mean | 9.90 | 8.71 | 12.04 | 8.74 |  | 10.25 | 8.84 | 9.93 | 8.05 |  | 13.57 | 7.39 | 11.48 | 6.48 |
|  |  | Group | a | a | a | b |  | a | a | a | a |  | a | b | a | b |
|  |  | SD | 1.11 | 1.42 | 0.95 | 1.40 |  | 0.14 | 1.58 | 0.85 | 0.86 |  | 1.42 | 1.45 | 1.44 | 1.81 |
|  | 7 | Mean | 18.18 | 14.77 | 18.16 | 12.30 |  | 16.82 | 12.43 | 13.32 | 11.66 |  | 17.81 | 12.81 | 16.46 | 11.13 |
|  |  | Group | a | a | a | b |  | a | b | a | a |  | a | b | a | b |
|  |  | SD | 1.38 | 2.55 | 3.05 | 1.23 |  | 0.61 | 0.92 | 0.72 | 1.01 |  | 2.08 | 1.81 | 1.76 | 2.03 |
|  | 14 | Mean | 23.62 | 26.41 | 23.45 | 17.96 |  | 20.55 | 20.04 | 17.00 | 15.57 |  | 27.17 | 20.80 | 21.65 | 14.92 |
|  |  | Group | a | a | a | a |  | a | a | a | a |  | a | a | a | a |
|  |  | SD | 0.56 | 2.39 | 3.19 | 1.64 |  | 1.00 | 2.27 | 1.06 | 4.04 |  | 0.99 | 5.04 | 4.36 | 3.52 |
|  | 21 | Mean | 31.75 | 31.54 | 22.20 | 20.79 |  | 28.72 | 26.83 | 16.51 | 16.21 |  | 30.64 | 26.00 | 22.47 | 18.34 |
|  |  | Group | a | a | a | a |  | a | a | a | a |  | a | a | a | a |
|  |  | SD | 1.20 | 3.35 | 2.95 | 2.94 |  | 1.07 | 2.47 | 1.60 | 4.11 |  | 0.77 | 3.66 | 3.39 | 6.33 |
|  | 28 | Mean | 35.84 | 38.78 | 25.07 | 26.77 |  | 28.83 | 30.41 | 22.54 | 20.84 |  | 37.56 | 28.20 | 26.05 | 21.62 |
|  |  | Group | a | a | a | a |  | a | a | a | a |  | a | b | a | a |
|  |  | SD | 2.83 | 4.54 | 3.41 | 2.33 |  | 0.71 | 3.04 | 1.57 | 3.06 |  | 2.13 | 3.77 | 1.60 | 6.18 |
|  | 35 | Mean | 43.23 | 47.77 | 34.67 | 34.49 |  | 33.27 | 34.71 | 25.85 | 25.83 |  | 40.25 | 32.32 | 27.60 | 24.00 |
|  |  | Group | b | a | a | a |  | a | a | a | a |  | a | a | a | a |
|  |  | SD | 2.08 | 1.61 | 2.15 | 5.45 |  | 1.11 | 2.67 | 1.20 | 2.65 |  | 5.04 | 1.97 | 2.94 | 4.26 |
|  | 42 | Mean | 49.31 | 55.91 | 42.74 | 41.28 |  | 37.60 | 39.92 | 28.04 | 28.06 |  | 41.40 | 38.72 | 31.31 | 26.28 |
|  |  | Group | b | a | a | a |  | a | a | a | a |  | a | a | a | a |
|  |  | SD | 3.76 | 0.25 | 0.97 | 1.44 |  | 1.60 | 1.86 | 1.03 | 2.85 |  | 1.15 | 3.84 | 1.54 | 7.52 |
|  | 56 | Mean | 55.82 | 62.30 | 49.48 | 46.68 |  | 37.26 | 40.03 | 28.77 | 28.23 |  | 41.21 | 44.00 | 34.08 | 30.15 |
|  |  | Group | b | a | a | a |  | a | a | a | a |  | a | a | a | a |
|  |  | SD | 2.60 | 1.64 | 1.90 | 3.34 |  | 3.22 | 2.21 | 2.14 | 4.16 |  | 1.31 | 5.49 | 4.02 | 8.02 |
|  | Harvest | Mean | 55.20 | 62.51 | 50.27 | 45.84 |  | 36.17 | 38.00 | 30.44 | 25.74 |  | 42.42 | 45.02 | 36.21 | 28.55 |
|  |  | Group | a | a | a | a |  | a | a | a | a |  | a | a | a | a |
|  |  | SD | 2.47 | 4.78 | 5.15 | 4.26 |  | 2.27 | 3.46 | 3.20 | 2.90 |  | 1.67 | 3.42 | 5.92 | 3.23 |
|  |  |  |  |  |  |  |  |  |  |  |  |  |  |  |  |  |
| Chlorophyll Content Index | -7 | Mean | 1.68 | 1.45 | 1.70 | 1.39 |  | 1.64 | 1.34 | 1.59 | 1.30 |  | 1.91 | 2.24 | 1.72 | 1.32 |
|  |  | Group | a | a | a | b |  | a | b | a | b |  | a | a | a | b |
|  |  | SD | 0.17 | 0.02 | 0.08 | 0.15 |  | 0.12 | 0.04 | 0.15 | 0.09 |  | 0.16 | 1.45 | 0.18 | 0.10 |
|  | 0 | Mean | 2.33 | 1.86 | 2.26 | 1.86 |  | 2.15 | 1.80 | 2.08 | 1.75 |  | 2.36 | 1.77 | 2.60 | 1.66 |
|  |  | Group | a | a | a | a |  | a | b | a | a |  | a | b | a | b |
|  |  | SD | 0.27 | 0.31 | 0.22 | 0.23 |  | 0.11 | 0.10 | 0.28 | 0.08 |  | 0.10 | 0.25 | 0.30 | 0.14 |
|  | 7 | Mean | 7.76 | 4.40 | 6.45 | 2.77 |  | 5.29 | 3.25 | 3.59 | 2.40 |  | 6.10 | 2.72 | 4.33 | 2.53 |
|  |  | Group | a | a | a | a |  | a | a | a | a |  | a | a | a | b |
|  |  | SD | 2.29 | 1.12 | 2.95 | 0.39 |  | 1.74 | 1.39 | 0.72 | 0.25 |  | 2.34 | 0.78 | 0.48 | 0.70 |
|  | 14 | Mean | 15.77 | 15.30 | 12.57 | 8.20 |  | 10.60 | 7.84 | 4.97 | 4.93 |  | 17.83 | 9.59 | 7.97 | 4.23 |
|  |  | Group | a | a | a | a |  | a | a | a | a |  | a | b | a | a |
|  |  | SD | 1.05 | 1.92 | 5.13 | 2.97 |  | 0.98 | 2.92 | 1.25 | 0.58 |  | 1.40 | 4.56 | 1.90 | 1.78 |
|  | 21 | Mean | 22.11 | 21.15 | 15.60 | 14.18 |  | 13.62 | 10.35 | 7.15 | 5.90 |  | 20.39 | 10.93 | 11.22 | 6.74 |
|  |  | Group | a | a | a | a |  | a | a | a | a |  | a | a | a | a |
|  |  | SD | 1.38 | 3.22 | 4.01 | 4.00 |  | 3.39 | 1.19 | 0.77 | 2.39 |  | 4.31 | 6.44 | 3.23 | 3.76 |
|  | 28 | Mean | 25.26 | 27.64 | 23.51 | 18.82 |  | 20.31 | 18.17 | 12.11 | 11.71 |  | 24.26 | 14.96 | 17.40 | 12.94 |
|  |  | Group | a | a | a | a |  | a | a | a | a |  | a | a | a | a |
|  |  | SD | 3.55 | 3.56 | 3.35 | 2.67 |  | 2.95 | 2.78 | 2.54 | 2.30 |  | 3.87 | 9.19 | 6.41 | 7.24 |
|  | 35 | Mean | 24.72 | 30.76 | 28.18 | 26.91 |  | 24.08 | 20.05 | 18.39 | 16.81 |  | 26.79 | 25.19 | 23.10 | 15.95 |
|  |  | Group | b | a | a | a |  | a | a | a | a |  | a | a | a | a |
|  |  | SD | 3.42 | 1.16 | 2.38 | 3.89 |  | 3.83 | 4.26 | 2.18 | 5.03 |  | 3.32 | 5.72 | 7.33 | 6.91 |
|  | 42 | Mean | 28.17 | 31.50 | 29.50 | 29.95 |  | 24.00 | 23.44 | 19.74 | 18.28 |  | 25.18 | 26.08 | 25.58 | 18.16 |
|  |  | Group | a | a | a | a |  | a | a | a | a |  | a | a | a | a |
|  |  | SD | 1.53 | 1.95 | 1.63 | 1.77 |  | 2.51 | 3.01 | 1.26 | 2.01 |  | 1.87 | 1.25 | 4.55 | 9.35 |
|  | 56 | Mean | 23.68 | 26.04 | 32.15 | 28.83 |  | 19.97 | 20.79 | 20.65 | 22.81 |  | 23.74 | 23.71 | 22.21 | 21.53 |
|  |  | Group | a | a | a | b |  | a | a | a | a |  | a | a | a | a |
|  |  | SD | 0.86 | 1.66 | 0.65 | 1.49 |  | 0.85 | 3.06 | 1.41 | 4.90 |  | 1.96 | 3.02 | 4.76 | 5.39 |
|  | Harvest | Mean | 24.19 | 24.51 | 27.37 | 26.19 |  | 17.65 | 18.50 | 20.66 | 17.49 |  | 21.93 | 19.74 | 18.89 | 18.79 |
|  |  | Group | a | a | a | a |  | a | a | a | a |  | a | b | a | a |
|  |  | SD | 0.73 | 3.22 | 3.03 | 3.52 |  | 1.51 | 3.07 | 3.53 | 2.38 |  | 0.64 | 1.01 | 4.31 | 1.32 |
| Leaf Temperature [°C] |  |  |  |  |  |  |  |  |  |  |  |  |  |  |  |  |
|  | -7 | Mean | 22.83 | 22.24 | 23.49 | 23.13 |  | 22.18 | 22.33 | 22.72 | 23.15 |  | 22.45 | 22.84 | 22.95 | 22.85 |
|  |  | Group | a | a | a | a |  | a | a | a | a |  | a | a | a | a |
|  |  | SD | 0.62 | 0.30 | 1.17 | 0.65 |  | 0.68 | 0.74 | 0.76 | 0.92 |  | 0.55 | 0.12 | 0.44 | 1.93 |
|  | 0 | Mean | 23.31 | 22.53 | 23.11 | 23.31 |  | 22.73 | 21.58 | 23.05 | 23.33 |  | 21.94 | 21.97 | 23.20 | 23.20 |
|  |  | Group | a | a | a | a |  | a | a | a | a |  | a | a | a | a |
|  |  | SD | 2.87 | 0.18 | 0.13 | 0.38 |  | 0.15 | 1.35 | 0.52 | 0.22 |  | 0.36 | 0.25 | 0.76 | 0.48 |
|  | 7 | Mean | 21.42 | 22.11 | 21.95 | 22.44 |  | 21.63 | 21.45 | 22.72 | 22.48 |  | 21.63 | 21.62 | 22.79 | 23.00 |
|  |  | Group | a | a | a | a |  | a | a | a | a |  | a | a | a | a |
|  |  | SD | 0.32 | 0.62 | 1.11 | 0.18 |  | 0.93 | 0.91 | 0.70 | 0.57 |  | 0.93 | 0.82 | 0.74 | 1.95 |
|  | 14 | Mean | 22.12 | 21.82 | 23.00 | 22.93 |  | 22.26 | 21.76 | 22.36 | 23.63 |  | 22.12 | 22.69 | 23.01 | 23.31 |
|  |  | Group | a | a | a | a |  | a | a | a | a |  | a | a | a | a |
|  |  | SD | 0.60 | 0.21 | 1.10 | 0.54 |  | 0.50 | 0.46 | 0.47 | 0.76 |  | 0.21 | 0.89 | 1.35 | 0.20 |
|  | 21 | Mean | 20.46 | 21.49 | 20.75 | 21.04 |  | 21.48 | 21.04 | 20.48 | 21.26 |  | 20.69 | 21.52 | 21.98 | 21.15 |
|  |  | Group | a | a | a | a |  | a | a | a | a |  | a | a | a | a |
|  |  | SD | 0.96 | 0.55 | 1.12 | 0.46 |  | 0.92 | 1.00 | 0.53 | 0.64 |  | 1.14 | 1.61 | 1.69 | 1.07 |
|  | 28 | Mean | 21.39 | 21.59 | 21.62 | 21.38 |  | 22.05 | 21.48 | 21.41 | 21.91 |  | 21.45 | 21.93 | 22.11 | 25.86 |
|  |  | Group | a | a | a | a |  | a | a | a | a |  | a | a | a | a |
|  |  | SD | 0.75 | 0.61 | 0.42 | 0.11 |  | 0.70 | 0.86 | 0.54 | 0.10 |  | 0.66 | 1.13 | 1.47 | 6.22 |
|  | 35 | Mean | 22.72 | 23.12 | 22.38 | 22.68 |  | 23.31 | 22.77 | 22.35 | 22.74 |  | 22.51 | 23.13 | 23.00 | 22.80 |
|  |  | Group | a | a | a | a |  | a | a | a | a |  | a | a | a | a |
|  |  | SD | 0.99 | 0.48 | 0.62 | 0.23 |  | 0.78 | 1.21 | 0.68 | 0.42 |  | 0.51 | 1.17 | 1.34 | 1.38 |
|  | 42 | Mean | 22.44 | 22.10 | 25.89 | 21.96 |  | 22.20 | 26.65 | 22.24 | 22.39 |  | 22.00 | 22.78 | 22.41 | 22.02 |
|  |  | Group | a | a | a | a |  | a | a | a | a |  | a | a | a | a |
|  |  | SD | 0.34 | 0.51 | 7.25 | 0.36 |  | 0.80 | 7.41 | 0.88 | 1.00 |  | 0.11 | 0.62 | 1.26 | 0.93 |
|  | 56 | Mean | 22.96 | 23.14 | 22.61 | 22.63 |  | 23.16 | 22.78 | 22.55 | 22.59 |  | 22.93 | 23.10 | 22.79 | 22.07 |
|  |  | Group | a | a | a | a |  | a | a | a | a |  | a | a | a | a |
|  |  | SD | 0.24 | 0.64 | 0.36 | 0.39 |  | 0.74 | 0.65 | 0.46 | 0.46 |  | 0.47 | 0.72 | 0.66 | 0.28 |
|  | Harvest | Mean | 24.86 | 23.62 | 23.89 | 23.48 |  | 23.86 | 23.63 | 23.39 | 24.07 |  | 23.31 | 23.89 | 25.11 | 22.27 |
|  |  | Group | a | a | a | a |  | a | a | a | a |  | a | a | a | a |
|  |  | SD | 1.53 | 0.97 | 1.15 | 1.36 |  | 0.63 | 1.51 | 0.94 | 1.79 |  | 0.97 | 0.72 | 1.61 | 2.18 |
