# Supplementary material for: Effect of a QTL on wheat chromosome 5B associated with enhanced root dry mass on transpiration and nitrogen uptake under contrasting drought scenarios in wheat
Source: BMC Plant Biol. 2024 Feb 2;24:83. doi: 10.1186/s12870-024-04756-8 (PMC10835935; doi:10.1186/s12870-024-04756-8)
Supplement: Supplementary file 6 — Additional file 6. Descriptive statistics for cumulative transpiration [L] and daily transpiration [ml] recorded in experiment 1 from -84 DAH until 35 DAH [file 12870_2024_4756_MOESM6_ESM.docx]

Additional file 6: Descriptive statistics for cumulative transpiration [L] and daily transpiration [ml] recorded in experiment 1 from -84 DAH until 35 DAH

| Trait | DAT | Treatment | Well-Watered | | | |  | Drought Scenario 1 | | | |  | Drought Scenario 2 | | | |
| --- | --- | --- | --- | --- | --- | --- | --- | --- | --- | --- | --- | --- | --- | --- | --- | --- |
|  |  | Genotype | Elixer | Genius | Leandrus | Ning 0604 |  | Elixer | Genius | Leandrus | Ning 0604 |  | Elixer | Genius | Leandrus | Ning 0604 |
| Cumulative Transpiration [L] | -84 | Mean | 0.50 | 0.32 | 0.93 | NA |  | 0.42 | 0.32 | 0.93 | NA |  | 0.41 | 0.37 | 0.96 | NA |
|  |  | Group | b | c | a | NA |  | b | c | a | NA |  | ab | b | a | NA |
|  |  | SD | 0.07 | 0.11 | 0.10 | NA |  | 0.11 | 0.05 | 0.09 | NA |  | 0.12 | 0.06 | 0.35 | NA |
|  | -77 | Mean | 1.08 | 0.69 | 1.94 | NA |  | 1.02 | 0.73 | 1.87 | NA |  | 1.00 | 0.82 | 1.98 | NA |
|  |  | Group | b | c | a | NA |  | b | c | a | NA |  | b | b | a | NA |
|  |  | SD | 0.17 | 0.11 | 0.26 | NA |  | 0.24 | 0.06 | 0.19 | NA |  | 0.21 | 0.12 | 0.58 | NA |
|  | -70 | Mean | 2.24 | 1.59 | 2.86 | NA |  | 2.18 | 1.64 | 2.74 | NA |  | 2.24 | 1.91 | 2.98 | NA |
|  |  | Group | a | b | a | NA |  | b | c | a | NA |  | b | b | a | NA |
|  |  | SD | 0.34 | 0.26 | 0.38 | NA |  | 0.48 | 0.14 | 0.34 | NA |  | 0.37 | 0.18 | 0.72 | NA |
|  | -63 | Mean | 3.34 | 2.47 | 4.34 | 0.52 |  | 3.25 | 2.48 | 4.28 | 0.52 |  | 3.48 | 2.94 | 4.66 | 0.53 |
|  |  | Group | ab | b | a | c |  | a | b | a | c |  | a | a | a | b |
|  |  | SD | 0.42 | 0.49 | 0.45 | 0.10 |  | 0.72 | 0.17 | 0.60 | 0.14 |  | 0.59 | 0.15 | 0.90 | 0.12 |
|  | -56 | Mean | 4.64 | 3.47 | 6.11 | 1.86 |  | 4.53 | 3.42 | 6.15 | 1.67 |  | 4.77 | 4.07 | 6.62 | 1.86 |
|  |  | Group | ab | bc | a | c |  | ab | b | a | c |  | ab | b | a | c |
|  |  | SD | 0.55 | 0.83 | 0.66 | 0.23 |  | 1.01 | 0.27 | 1.06 | 0.14 |  | 0.83 | 0.12 | 1.06 | 0.11 |
|  | -49 | Mean | 6.38 | 5.00 | 9.06 | 3.92 |  | 6.30 | 4.93 | 9.10 | 3.69 |  | 6.82 | 5.81 | 9.72 | 3.92 |
|  |  | Group | ab | b | a | b |  | b | bc | a | c |  | ab | bc | a | c |
|  |  | SD | 0.84 | 1.30 | 0.94 | 0.58 |  | 1.48 | 0.51 | 1.39 | 0.41 |  | 1.25 | 0.42 | 1.12 | 0.37 |
|  | -42 | Mean | 9.50 | 7.90 | 12.55 | 7.07 |  | 9.36 | 7.88 | 12.58 | 6.71 |  | 10.18 | 8.81 | 13.31 | 7.02 |
|  |  | Group | ab | b | a | b |  | b | bc | a | c |  | ab | bc | a | c |
|  |  | SD | 1.44 | 1.79 | 1.34 | 0.98 |  | 2.08 | 0.96 | 1.72 | 0.71 |  | 1.98 | 1.02 | 1.16 | 0.85 |
|  | -35 | Mean | 13.23 | 11.43 | 16.14 | 10.39 |  | 12.95 | 11.34 | 16.00 | 9.93 |  | 14.38 | 12.47 | 17.04 | 10.26 |
|  |  | Group | ab | ab | a | b |  | a | ab | a | b |  | a | ab | a | b |
|  |  | SD | 1.93 | 2.04 | 1.71 | 1.34 |  | 2.64 | 1.43 | 1.88 | 0.91 |  | 2.75 | 1.69 | 1.20 | 1.14 |
|  | -28 | Mean | 17.13 | 14.87 | 19.84 | 14.21 |  | 16.65 | 14.80 | 19.46 | 13.58 |  | 18.71 | 16.00 | 20.65 | 13.95 |
|  |  | Group | a | a | a | a |  | a | a | a | b |  | a | ab | a | b |
|  |  | SD | 2.63 | 2.17 | 2.07 | 2.06 |  | 3.29 | 2.03 | 2.01 | 1.28 |  | 3.69 | 2.65 | 1.19 | 1.54 |
|  | -21 | Mean | 20.64 | 17.73 | 23.07 | 18.86 |  | 19.97 | 17.66 | 22.70 | 17.89 |  | 22.47 | 18.71 | 24.09 | 18.20 |
|  |  | Group | a | b | a | a |  | a | a | a | a |  | a | a | a | a |
|  |  | SD | 3.17 | 2.26 | 2.53 | 2.99 |  | 3.75 | 2.49 | 2.24 | 1.62 |  | 4.31 | 3.35 | 1.28 | 1.76 |
|  | -14 | Mean | 24.06 | 20.22 | 26.56 | 23.72 |  | 23.32 | 20.14 | 26.13 | 22.66 |  | 26.24 | 21.26 | 27.69 | 22.79 |
|  |  | Group | a | b | a | a |  | a | a | a | a |  | a | a | a | a |
|  |  | SD | 3.90 | 2.32 | 3.01 | 3.94 |  | 4.34 | 3.10 | 2.60 | 1.92 |  | 5.01 | 4.31 | 1.50 | 1.93 |
|  | -7 | Mean | 27.39 | 22.86 | 30.06 | 28.40 |  | 26.48 | 22.81 | 29.68 | 27.40 |  | 29.98 | 23.93 | 31.33 | 27.24 |
|  |  | Group | a | b | a | a |  | a | a | a | a |  | a | a | a | a |
|  |  | SD | 4.58 | 2.43 | 3.26 | 4.98 |  | 5.05 | 3.90 | 2.99 | 2.45 |  | 5.66 | 5.28 | 1.65 | 2.11 |
|  | HD | Mean | 31.20 | 25.32 | 33.29 | 32.06 |  | 30.47 | 25.37 | 32.75 | 30.97 |  | 33.85 | 26.60 | 34.47 | 30.65 |
|  |  | Group | a | b | a | a |  | a | a | a | a |  | a | a | a | a |
|  |  | SD | 4.97 | 2.53 | 3.49 | 5.44 |  | 5.38 | 4.34 | 3.30 | 2.74 |  | 5.90 | 5.77 | 1.61 | 2.20 |
|  | 7 | Mean | 34.77 | 27.88 | 36.25 | 35.65 |  | 32.55 | 26.94 | 35.53 | 32.79 |  | 37.67 | 29.67 | 37.78 | 33.05 |
|  |  | Group | a | b | a | a |  | a | a | a | a |  | a | a | a | a |
|  |  | SD | 5.19 | 2.73 | 3.43 | 6.27 |  | 5.71 | 4.78 | 3.61 | 3.04 |  | 6.15 | 6.27 | 1.57 | 2.30 |
|  | 14 | Mean | 38.20 | 30.38 | 38.88 | 38.73 |  | 33.14 | 27.46 | 37.45 | 33.08 |  | 40.01 | 31.70 | 40.90 | 34.46 |
|  |  | Group | a | b | a | a |  | a | a | a | a |  | a | a | a | a |
|  |  | SD | 5.33 | 2.93 | 3.30 | 6.98 |  | 5.72 | 4.81 | 3.63 | 3.08 |  | 5.90 | 6.33 | 1.64 | 2.24 |
|  | 21 | Mean | 40.68 | 32.36 | 40.90 | 41.09 |  | 33.67 | 27.85 | 38.88 | 33.52 |  | 41.24 | 33.16 | 42.34 | 35.22 |
|  |  | Group | a | b | a | a |  | a | a | a | a |  | a | b | a | b |
|  |  | SD | 5.40 | 2.97 | 3.15 | 7.36 |  | 5.78 | 4.79 | 3.55 | 3.25 |  | 5.67 | 6.38 | 1.64 | 2.21 |
|  | 28 | Mean | 42.64 | 33.74 | 42.10 | 42.70 |  | 34.27 | 28.21 | 39.64 | 33.94 |  | 42.14 | 34.05 | 42.86 | 35.63 |
|  |  | Group | a | b | a | a |  | a | a | a | NA |  | a | b | a | b |
|  |  | SD | 5.27 | 2.92 | 3.22 | 8.00 |  | 5.87 | 4.73 | 3.41 | NA |  | 5.35 | 6.34 | 1.54 | 2.26 |
|  | 35 | Mean | 44.00 | 34.77 | NA | NA |  | 34.68 | 28.45 | NA | NA |  | 42.71 | 34.39 | NA | NA |
|  |  | Group | a | b | NA | NA |  | a | a | NA | NA |  | a | b | NA | NA |
|  |  | SD | 5.05 | 2.98 | NA | NA |  | 5.89 | 4.66 | NA | NA |  | 4.97 | 6.20 | NA | NA |
|  |  |  |  |  |  |  |  |  |  |  |  |  |  |  |  |  |
| Daily Transpiration [ml] | -84 | Mean | 53.67 | 33.67 | 138.83 | NA |  | 51.33 | 34.33 | 145.67 | NA |  | 51.67 | 37.67 | 143.50 | NA |
|  |  | Group | a | a | a | NA |  | a | b | ab | NA |  | a | a | a | NA |
|  |  | SD | 18.15 | 4.51 | 19.66 | NA |  | 20.86 | 7.20 | 3.43 | NA |  | 12.66 | 20.84 | 25.00 | NA |
|  | -77 | Mean | 147.17 | 43.50 | 167.42 | NA |  | 152.50 | 42.00 | 153.75 | NA |  | 147.17 | 53.83 | 174.75 | NA |
|  |  | Group | b | b | a | NA |  | b | b | a | NA |  | b | b | a | NA |
|  |  | SD | 5.51 | 8.54 | 34.24 | NA |  | 18.10 | 13.78 | 32.56 | NA |  | 11.15 | 3.06 | 55.38 | NA |
|  | -70 | Mean | 208.08 | 143.42 | 155.75 | NA |  | 193.75 | 139.42 | 145.42 | NA |  | 217.08 | 178.42 | 165.75 | NA |
|  |  | Group | a | b | b | NA |  | a | b | ab | NA |  | a | a | a | NA |
|  |  | SD | 32.81 | 21.01 | 20.07 | NA |  | 60.16 | 16.02 | 34.43 | NA |  | 45.72 | 30.35 | 33.18 | NA |
|  | -63 | Mean | 176.42 | 164.75 | 237.25 | 164.83 |  | 180.08 | 145.92 | 252.95 | 169.50 |  | 208.08 | 190.42 | 259.08 | 165.50 |
|  |  | Group | a | a | b | a |  | ab | b | c | a |  | a | a | b | a |
|  |  | SD | 28.02 | 48.66 | 21.92 | 23.97 |  | 50.37 | 25.68 | 62.49 | 29.86 |  | 49.33 | 21.55 | 21.39 | 14.84 |
|  | -56 | Mean | 177.42 | 71.08 | 365.83 | 221.08 |  | 187.42 | 49.92 | 374.50 | 210.08 |  | 110.42 | 79.75 | 407.17 | 213.42 |
|  |  | Group | b | b | a | b |  | b | b | a | b |  | b | b | a | b |
|  |  | SD | 32.72 | 59.55 | 46.32 | 24.83 |  | 60.75 | 43.52 | 67.65 | 15.34 |  | 27.21 | 42.67 | 23.44 | 23.09 |
|  | -49 | Mean | 419.83 | 393.17 | 452.58 | 316.25 |  | 423.67 | 414.33 | 460.42 | 301.75 |  | 473.17 | 433.50 | 473.92 | 301.58 |
|  |  | Group | a | ab | b | a |  | a | a | b | a |  | a | a | b | ab |
|  |  | SD | 75.18 | 96.57 | 37.45 | 80.16 |  | 89.93 | 57.98 | 36.82 | 62.72 |  | 82.55 | 80.07 | 25.32 | 75.74 |
|  | -42 | Mean | 497.92 | 464.25 | 603.67 | 469.42 |  | 499.75 | 403.58 | 592.50 | 449.42 |  | 445.25 | 400.92 | 646.67 | 453.75 |
|  |  | Group | ab | ab | a | b |  | a | a | a | b |  | a | ab | a | b |
|  |  | SD | 81.59 | 77.36 | 81.65 | 69.17 |  | 115.57 | 80.83 | 72.88 | 51.48 |  | 137.20 | 130.93 | 10.54 | 55.51 |
|  | -35 | Mean | 676.67 | 580.67 | 497.08 | 562.08 |  | 647.50 | 503.83 | 387.42 | 527.42 |  | 749.33 | 547.67 | 428.75 | 533.75 |
|  |  | Group | a | a | b | c |  | a | a | b | c |  | a | ab | bc | c |
|  |  | SD | 109.42 | 39.12 | 48.76 | 105.31 |  | 130.29 | 117.01 | 53.81 | 63.25 |  | 192.58 | 185.27 | 70.62 | 64.86 |
|  | -28 | Mean | 443.75 | 475.42 | 519.92 | 582.08 |  | 433.92 | 452.25 | 424.75 | 532.75 |  | 497.08 | 412.75 | 449.92 | 530.42 |
|  |  | Group | ab | b | b | a |  | b | b | b | a |  | a | a | a | a |
|  |  | SD | 95.52 | 29.74 | 112.17 | 138.44 |  | 88.69 | 91.46 | 69.01 | 59.27 |  | 98.43 | 138.57 | 25.42 | 47.63 |
|  | -21 | Mean | 489.58 | 355.58 | 474.25 | 734.17 |  | 442.58 | 356.92 | 482.08 | 687.83 |  | 542.92 | 426.58 | 519.25 | 677.17 |
|  |  | Group | a | ab | b | ab |  | a | a | b | ab |  | a | a | a | a |
|  |  | SD | 112.67 | 8.14 | 55.83 | 69.40 |  | 130.47 | 120.12 | 58.44 | 52.79 |  | 183.04 | 203.57 | 41.61 | 22.50 |
|  | -14 | Mean | 519.25 | 333.25 | 496.92 | 681.58 |  | 486.42 | 276.42 | 490.92 | 689.92 |  | 555.92 | 273.58 | 540.92 | 665.92 |
|  |  | Group | bc | c | b | a |  | c | c | b | a |  | a | b | a | a |
|  |  | SD | 86.79 | 45.13 | 51.38 | 168.93 |  | 94.69 | 75.07 | 96.84 | 113.34 |  | 64.13 | 104.70 | 51.86 | 73.32 |
|  | -7 | Mean | 430.25 | 362.25 | 430.83 | 567.17 |  | 438.75 | 419.92 | 431.50 | 583.83 |  | 525.58 | 428.92 | 423.17 | 543.83 |
|  |  | Group | b | b | b | a |  | b | b | b | a |  | a | b | b | a |
|  |  | SD | 90.04 | 42.76 | 47.08 | 54.52 |  | 90.77 | 113.96 | 95.70 | 57.29 |  | 67.49 | 116.72 | 23.46 | 20.66 |
|  | HD | Mean | 514.75 | 377.08 | 485.75 | 577.00 |  | 559.42 | 409.50 | 422.83 | 566.50 |  | 544.08 | 449.42 | 469.33 | 556.67 |
|  |  | Group | a | b | a | a |  | a | b | b | a |  | a | b | b | a |
|  |  | SD | 56.19 | 50.83 | 29.87 | 70.19 |  | 60.23 | 64.70 | 65.55 | 42.79 |  | 48.25 | 68.66 | 29.86 | 17.23 |
|  | 7 | Mean | 458.75 | 377.75 | 415.42 | 404.33 |  | 143.08 | 94.42 | 315.42 | 142.60 |  | 4384.42 | 344.42 | 446.08 | 340.00 |
|  |  | Group | a | a | a | a |  | b | b | a | b |  | a | b | a | b |
|  |  | SD | 22.34 | 58.90 | 12.66 | 85.86 |  | 29.69 | 15.44 | 35.40 | 28.28 |  | 29.02 | 20.60 | 36.25 | 13.80 |
|  | 14 | Mean | 391.75 | 366.42 | 352.92 | 437.58 |  | 108.58 | 96.42 | 231.58 | 48.42 |  | 222.42 | 282.08 | 433.58 | 181.25 |
|  |  | Group | a | a | a | a |  | b | b | a | c |  | b | b | a | b |
|  |  | SD | 12.17 | 24.70 | 37.43 | 98.08 |  | 36.39 | 11.67 | 16.07 | 33.60 |  | 73.57 | 7.37 | 16.77 | 10.26 |
|  | 21 | Mean | 362.92 | 225.92 | 245.92 | 280.25 |  | 72.75 | 51.08 | 156.25 | 87.80 |  | 128.58 | 145.58 | 157.58 | 79.67 |
|  |  | Group | a | b | b | b |  | b | b | a | b |  | a | a | a | a |
|  |  | SD | 10.97 | 27.47 | 107.06 | 76.77 |  | 23.48 | 12.81 | 30.84 | 37.28 |  | 41.00 | 27.15 | 28.10 | 16.52 |
|  | 28 | Mean | 213.25 | 158.58 | 153.42 | 250.08 |  | 91.58 | 47.25 | 64.67 | 61.80 |  | 78.25 | 90.58 | 58.50 | 40.75 |
|  |  | Group | a | a | a | a |  | a | a | a | NA |  | a | a | a | a |
|  |  | SD | 53.25 | 55.77 | 44.00 | 105.22 |  | 108.41 | NA | NA | NA |  | 75.18 | 49.89 | 103.91 | 45.21 |
|  | 35 | Mean | 156.08 | 138.08 | NA | 128.50 |  | 43.50 | 8.25 | NA | 15.00 |  | 40.42 | 44.42 | NA | NA |
|  |  | Group | a | a | NA | NA |  | a | a | NA | NA |  | a | a | NA | NA |
|  |  | SD | 11.37 | 40.20 | NA | NA |  | 29.09 | 6.86 | NA | NA |  | 58.05 | 47.37 | NA | NA |
