# Supplementary material for: Effect of a QTL on wheat chromosome 5B associated with enhanced root dry mass on transpiration and nitrogen uptake under contrasting drought scenarios in wheat
Source: BMC Plant Biol. 2024 Feb 2;24:83. doi: 10.1186/s12870-024-04756-8 (PMC10835935; doi:10.1186/s12870-024-04756-8)

**Additional file 9**: Nitrogen use efficiency of shoot dry mass under contrasting irrigation treatments in experiment 2. Colours represent the genetic background; different letters indicate significance (*p* < 0.005) between the mean values of the genotypes in the specific irrigation treatment according to the Tukey test. Error bars represent standard errors.


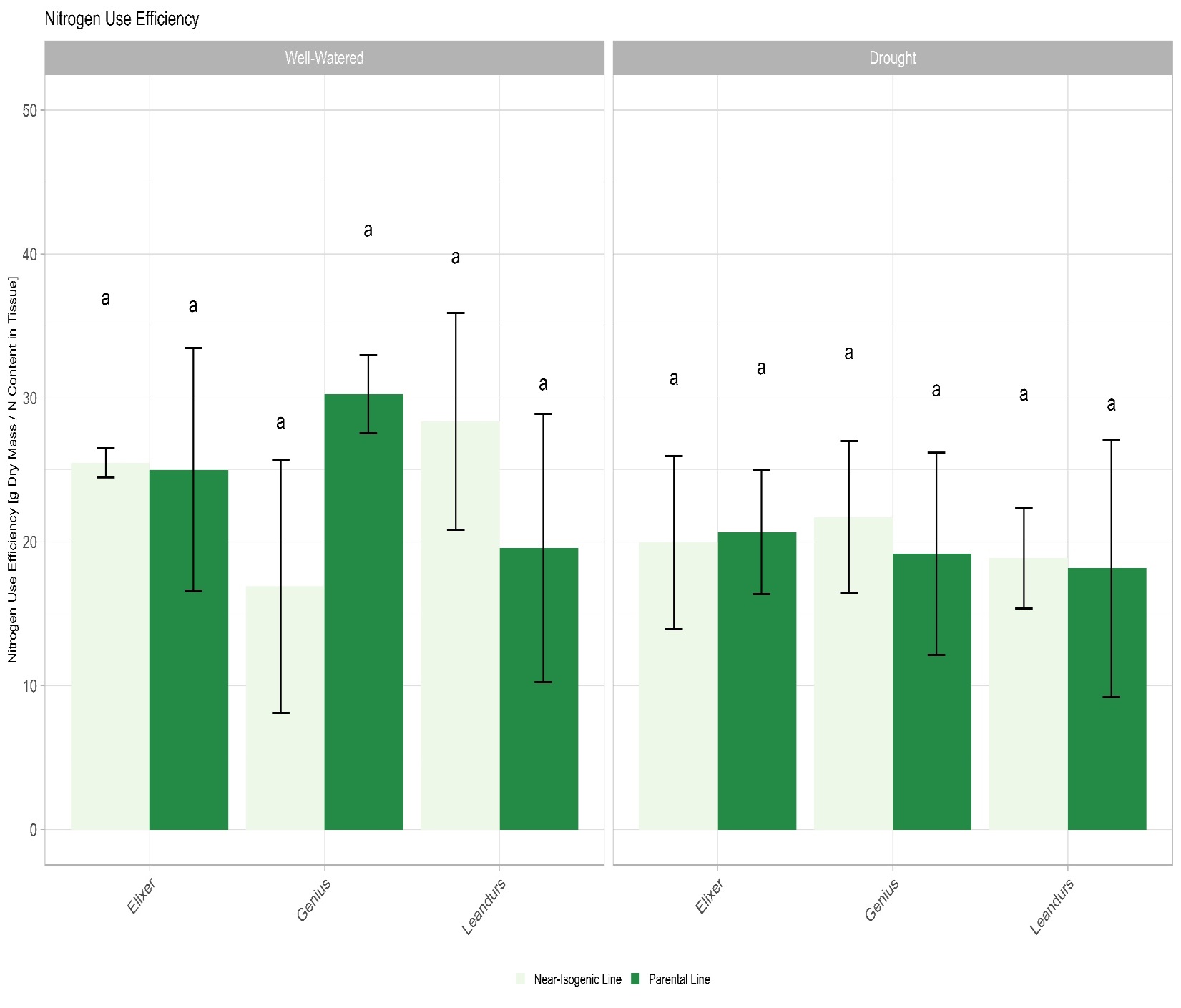

Supplement: Supplementary file 9 — Additional file 9. Nitrogen use efficiency of shoot dry mass under contrasting irrigation treatments in experiment 2. [file 12870_2024_4756_MOESM9_ESM.docx]
